# Effects of deworming on child and maternal health: a literature review and meta-analysis

**DOI:** 10.1186/s12889-017-4747-0

**Published:** 2017-11-07

**Authors:** Winter Maxwell Thayer, Adrienne Clermont, Neff Walker

**Affiliations:** 1 Department of Health Policy and Management, The Johns Hopkins Bloomberg School of Public Health, Baltimore, MD USA; 2 Department of International Health, The Johns Hopkins Bloomberg School of Public Health, Baltimore, MD USA

**Keywords:** Soil-transmitted helminth, Ascaris, Trichuris, Hookworm, Child health, Maternal health, Anemia, Stunting, Wasting, Malnutrition

## Abstract

**Background:**

Soil-transmitted helminth infections are widespread. Many studies have been published on the topic of deworming. The Lives Saved Tool (*LiST*) is a software package that uses a deterministic mathematical model to estimate the effect of scaling up interventions on maternal and child health outcomes. This review investigates the scope of available evidence for benefits of deworming treatments in order to inform a decision about possible inclusion of deworming as an intervention in *LiST*.

**Methods:**

We searched PubMed, the Cochrane Library, and Google Scholar. We included studies that reported pre/post data in children younger than 5 years or pregnant women for outcomes related to mortality and growth. We excluded studies that compared different anthelminthic treatments but did not include a placebo or non-treatment group, and those that did not report post-intervention outcomes. We categorized articles by treated population (children younger than 5 years and pregnant women), experimental versus observational, mass drug administration (MDA) versus treatment, and reported outcome.

**Results:**

We identified 58 relevant trials; 27 investigated children younger than 5 years and 11 investigated pregnant women; one reported on both children younger than 5 years and pregnant women. We conducted meta-analyses of relevant outcomes in children younger than 5 years.

**Conclusions:**

Deworming did not show consistent benefits for indicators of mortality, anemia, or growth in children younger than five or women of reproductive age. We do not recommend including the effect of deworming in the *LiST* model.

**Electronic supplementary material:**

The online version of this article (10.1186/s12889-017-4747-0) contains supplementary material, which is available to authorized users.

## Background

An estimated 1.3 billion people worldwide are currently infected with soil-transmitted helminths (STHs) [1]. There were an estimated 4.98 million years lived with disability [2] due to STH infections in 2010. The World Health Organization (WHO) recommends mass drug administration (MDA) of prophylactic chemotherapy once per year in all areas with greater than 20% helminth prevalence and twice per year in areas with over 50% prevalence [3]. MDA is the primary method of control for STH due to the expense of helminth diagnosis and low cost of deworming drugs. In 2014, over 400 million preschool-aged and school-aged children were targeted for deworming worldwide [4].

The Lives Saved Tool (*LiST*) is a software package that uses a deterministic mathematical model to estimate the effect of scaling up interventions on maternal and child health outcomes [5]. Intervention effect sizes are included in *LiST* based on regular reviews of the scientific literature. Many randomized controlled trials and meta-analyses have been published on the topic of deworming. The purpose of the current review is to investigate the scope of the available evidence for effects of STH treatment, and assess the possibility of including deworming as an intervention in *LiST*.

Benefits of deworming on child and maternal health outcomes rely on the assumption that deworming improves nutrition and hemoglobin levels, and thereby enhances wellbeing and reduces mortality. The primary outcomes in *LiST* are maternal, neonatal, and post-neonatal mortality and stillbirths. Research does not suggest a substantial impact of STH infection on child mortality; however, possible pathways in *LiST* for this relationship are via an impact on maternal anemia, perinatal mortality, diarrhea incidence in children, or the risk factors of anemia, stunting, wasting, or birth outcomes.

Meta-analyses by Cochrane researchers [6] and other researchers [7, 8] have investigated the impact of deworming on children 16 and younger, but have not separated out the effect on children younger than five. These analyses have disagreed about the studies that should be included, and the effect estimates and standard errors that should be used from some studies. Authors have argued that the failure of some meta-analyses to find an effect is an artifact of the dilution of the health benefits through the inclusion of uninfected and lightly infected individuals in MDA applications [9], and that the Cochrane methodology is inappropriate in the context of deworming [10]. That is, when both infected and uninfected individuals are treated the effect in infected individuals is not detected due to lack of statistical power. A recent meta-analysis of the effectiveness of deworming on reducing the prevalence of STH found that mass-drug administration led to a significantly greater reduction in *Ascaris* and hookworm prevalence, though there was no effect for *Trichuris* [11]. In addition, some have argued that single-dose mebendazole and albendazole for the treatment of hookworm and *Trichuris* have low efficacy [12], therefore including studies that administered only a single dose may dilute the effect of deworming. The most recent Cochrane review [6] performed a meta-regression to investigate the effect of length of follow-up on weight. This analysis suggested that meta-analyses of single- and multiple-dose studies should be analyzed separately as well as together to investigate the effects of each type of study. Multiple-dose studies are often conducted with a longer follow-up than single-dose studies, and they may reduce reinfection, which can be a confounding factor in single-dose studies. Authors have also suggested that multiple-dose studies may be of most relevance to policy makers because they are likely to capture longer-term benefits, while single-dose studies capture short-term effects [6].

Deworming has been a standard practice for children in many developing countries for at least 30 years. However, there is little evidence that deworming has a significant impact on child or maternal health at the population level. The most recent Cochrane review found no evidence of a beneficial effect of deworming programs on any health outcome measure in either pregnant women or children under the age of 16 [6]. However, a recent re-analysis of the studies that focused on weight gain in children who are dewormed did suggest that there could be an effect [7]. The differences between the two analyses stemmed from different interpretations of study results as well as differences in study inclusion criteria.

In the analyses presented here we will focus on the impact of de-worming on weight gain among children younger than five. We will also systematically vary the inclusion criteria as well as the different approaches to estimating effect sizes from the studies from the Cochrane review [6] and Croke 2016 analysis [7]. This approach will raise the possibility that we will increase the chance of type I errors, but also provide a more robust test of the Cochrane’s findings of no population level effects on child health.

Overall this paper will present three sets of results. First, we will provide an overview of the available studies that measure the impact of available studies that measure the impact of deworming on the health of children younger than five. This paper will focus on two outcomes; hemoglobin levels and weight gain. Analyses were also performed on an additional 15 outcomes, which can be found in the supplemental material (Additional file 1). Second, we will review the available literature on the effects of deworming among pregnant women. The review focuses on four outcomes with greater data availability and relevance to LiST (perinatal mortality, maternal anemia, maternal hemoglobin, and birth weight), but seven additional outcomes are reported in the supplemental material (Additional file 1). Finally, we will present a set of meta-analyses on the impact of deworming on weight in children under the age of five using various assumptions and analyses to test the robustness of the reported findings.

## Methods

This review assesses the impact of deworming interventions on maternal and child health outcomes. We compiled the available evidence for an impact of deworming on children younger than 5 years, school-aged children, and pregnant women for measures related to mortality, anemia, growth, and other outcomes. After searching the literature, we evaluated the quality of studies according to the Child Health Epidemiology Reference Group (CHERG) adaptation of the Grading of Recommendations, Assessments, and Development and Education (GRADE) criteria [13] of studies that recent Cochrane Reviews had not already assessed.

We searched PubMed, the Cochrane Library, and Google Scholar databases. We also searched the reference lists of identified articles and included relevant articles found therein. Our search terms included various combinations of *helminthiasis*, *helminth, deworming*, *mothers, maternal, child, mortality, anemia, infectious disease morbidity, stunting,* and *wasting* in each of the databases. We considered meta-analyses, randomized (cluster and individual) trials, quasi-randomized trials, repeated cross-sectional studies, longitudinal studies, and nonrandomized community-based studies for inclusion.

We included studies that reported pre/post data for outcomes including mortality, anemia and related measures (e.g., iron and hemoglobin levels), and anthropometric measures (e.g., height, weight, birthweight). Studies without a non-anthelminthic comparison group and studies that did not report post-intervention health outcomes were excluded. Studies with mixed populations (e.g., children and adults) that could not be disaggregated were excluded. Studies were classified as either “mass drug administration” (MDA) or “treatment” according to whether prophylaxis was administered indiscriminately to all participants or exclusively to individuals with identified helminth infections.

We abstracted data in studies of children younger than five about mortality, hemoglobin, anemia prevalence, serum ferritin, plasma albumin, weight, height, height-for-age, weight-for-age, weight-for-height, body mass index, mid-upper arm circumference, triceps skinfold thickness, blood xylose, fat excretion, nutrient excretion, serum retinol, cytokine response, α_1_-antichymotrypsin, intestinal permeability, infectious disease incidence, and immune response. In studies of women of reproductive age we abstracted data about perinatal mortality, maternal hemoglobin, maternal anemia, birthweight, infant anemia, child hemoglobin, iron deficiency, serum ferritin, adverse birth outcomes, congenital abnormalities, and infectious disease indicators.

Meta-analyses have been reported on children under 16 years old; however, to the best of our knowledge no meta-analyses have assessed the effect of deworming exclusively in children younger than 5 years. We considered randomized and quasi-randomized trials for meta-analyses on outcomes assessed in populations that primarily contained children younger than 5 years (see Additional file 1: Table S1). Meta-analyses were conducted on the outcomes of mortality, hemoglobin concentration, height, weight, HAZ, WAZ, WHZ, and MUAC. All analyses were conducted using *R* [14] with the *metafor* and *superheat* packages [15, 16]. We used estimates of differences from baseline to follow-up where available, and follow-up information when baseline data were not provided. We specified random-effect models with restricted maximum-likelihood estimators to account for unobserved heterogeneity. Residual heterogeneity was assessed with the *I* ^*2*^ statistic. Model robustness was checked with leave-one-out analyses. Due to disagreements about the appropriate estimates to include in meta-analyses of weight, we conducted meta-analyses with all possible combinations of disagreed-upon weight estimates.

Recent meta-analyses have investigated the effects of deworming women of reproductive age, so no meta-analyses were conducted for this population. We still included this population in the review because it is of specific interest to *LiST*, and to investigate to effects reported by different meta-analyses on the same outcome as well as observational studies that have not been included.

Some estimates of the effect of deworming children younger than five on weight were not available in the original study, but have been acquired by the authors of reviews and meta-analyses. Where available, we used these updated estimates.

## Results and discussion

We identified 1609 articles in PubMed, 390 in the Cochrane Library, and 10,600 in Google Scholar. After screening titles, 174 article abstracts were assessed, and 75 articles were included. Articles reported on 61 unique studies that were carried out in 28 different countries. Twenty-eight unique studies investigated children younger than 5 years, 22 investigated school-aged children, and 11 investigated women of reproductive age. Twelve studies included both children younger and older than 5 years. Six included children up to 6 years old, two included children up to seven, three included children up to eight, and one included children up to nine. The majority of participants in these studies appeared to be younger than 5 years, thus they are included with the results of children under five (see Additional file 1: Table S1). We identified nine trials with multiple reports; these reports are cited by the article where the relevant result was reported. All results are reported by the population that was treated, i.e., infant outcomes after maternal deworming are included with the results for pregnant women.

### Children younger than five

**Fig. 1:**
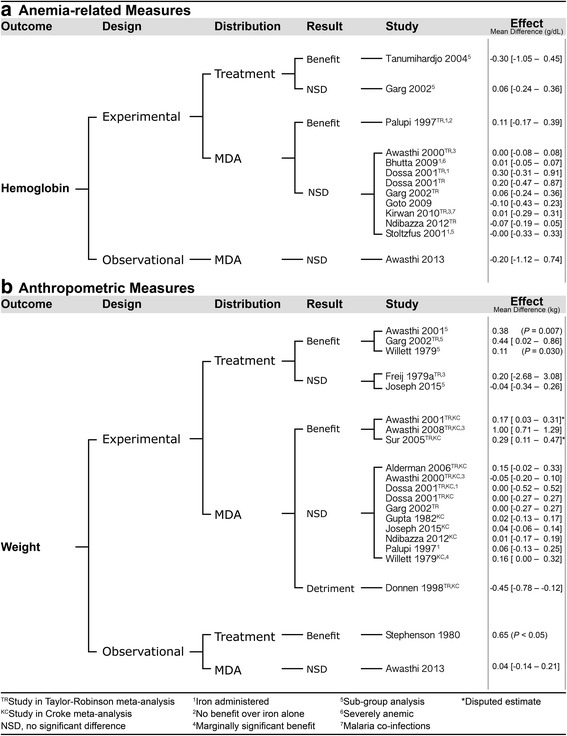
Effects of deworming children younger than 5 years. Identified evidence for anemia-related (panel **a**) and anthropometric measures (panel **b**) is grouped by design, distribution type, and result

We identified 28 studies of children younger than 5 years. The studies included 26 randomized (20 individual, 6 cluster) trials, one nonrandomized pre-post study, and one repeated cross-sectional study. Four trials were quasi-randomized [17–20]. One article (Tanumihardjo 2004) [21] reported two studies nested within one of the cluster-randomized trials; groups were randomized to the timing of deworming (one week before baseline measurement, at baseline, or after the follow-up measurements) and another group was measured before and after treatment but not randomized. One study reported two separate quasi-randomized trials (Freij 1979a and Friej 1979b) [19]. One cluster randomized trial reported the effects of deworming on mortality in over one million children, and additionally reported effects on other outcomes in children selected from within clusters without randomization [22]. One study (Goto 2009) used an effect of treatment on the treated analysis rather than an intention to treat analysis [23]. Twenty-two studies investigated MDA programs, six studies assessed the effect of deworming on infected children (see Fig. 1). We restrict the results reported here to hemoglobin and weight because these measures are of primary importance for our objective and a substantial amount of evidence has been reported. For additional health outcomes see the supplemental material (Additional file 1).

#### Children younger than five, anemia-related measures

One treatment trial [21] and eight MDA trials [17, 20, 24–29] investigated the impact of deworming on hemoglobin; one of these trials [26] conducted a sub-group analysis with only helminth-infected individuals. One study [22] reported observational results for hemoglobin. Tanumihardjo 2004 showed that children who received vitamin A and albendazole one week before baseline measurements had a significant improvement in hemoglobin at four-week follow-up (*n* = 21, 110 ± 8.6 vs. 121 ± 12.7, *P* = 0.004), while children who received albendazole one week after baseline (*n* = 19) and after follow-up measurements (*n* = 11) found no significant improvement. Palupi 1997 reported significantly greater gains in hemoglobin for participants randomized to iron supplementation only (*n* = 96) and deworming plus iron supplement (*n* = 95) groups compared to those in the placebo only (*n* = 98) group (*P* < 0.05); differences were not statistically significant between the iron supplement alone and with albendazole groups (*P =* 0.53). The other six studies did not show a significant difference between deworming and placebo groups.

#### Children younger than five, anthropometric measures

Thirteen MDA trials [17, 18, 25–28, 30–36] reported the effect of deworming on weight. Two trials [26, 34] conducted sub-group analyses of only helminth-infected individuals and one [31] conducted a sub-analysis on children whose mothers reported a history of *Ascaris* passage. One article [19] reported the results of a quasi-randomized treatment trial and a quasi-randomized MDA trial. One article reported a longitudinal study [37], and one article reported observational results [22]. Awasthi 2001 reported significantly greater mean weight gain in the treatment than the control groups, but the article reports conflicting standard error estimates that imply contradictory significance levels. In their abstract, Awasthi 2001 reports significantly greater weight gain in the albendazole and vitamin A clusters [*n* = 832, 3.22 kg, SE = 0.03] than the vitamin A alone clusters [*n* = 840, 3.04 kg, SE = 0.03, *P =* 0.01], in their text they report that weight gain in the control group was 3.05 kg, and at a later point in the text they report different standard deviations and standard errors (Treatment: SD = 2.03, SE = 0.26; Control: SD = 1.47, SE = 0.19). In this review, we have reported the benefits according to the authors’ interpretation that the results were significant. Awasthi 2001 also reports that the difference in weight gain was significantly greater in children that received albendazole plus vitamin A whose mother gave a history of *Ascaris* passage (*n* = 301, mean = 3.28 kg, SE = 0.17) than in children that received vitamin A alone whose mothers gave a history of *Ascaris* passage (*n* = 143, mean = 2.90 kg, SE = 0.17). Awasthi 2008 reported that children in urban slums randomized to receive albendazole every 6 months along with vitamin A supplementation and usual care (*n* = 25 slums, 1860 children) had greater mean weight gain than those in slums that were allocated to only vitamin A supplementation and usual care (*n* = 25 slums, 1852 children) at one year (mean = 1.57 kg, SE = 0.06 vs. 1.93 kg, SE = 0.08, mean difference = 0.36 kg, SE = 0.1, *P* < 0.001) and at 2 years (2.8 kg, SE = 0.1 vs. 3.8 kg, SE = 0.1, mean difference = 1.0 kg, SE = 0.15 *P* < 0.001; intra-class correlation coefficient = 0.17; 95% CI: 0.11 – 0.23)*.* Garg 2002 found no significant differences overall, but their sub-group analysis of helminth-infected individuals showed significantly greater mean weight gain for treated (*n* = 22) than untreated children (*n* = 20) [1.53 kg ± 0.15 vs. 1.09 kg ± 0.15, *P =* 0.04]. Sur 2005 showed a significantly greater mean increase in weight in the deworming group than the placebo group at three (*n*s = 345 and 340, *P* < 0.01), six (*n*s = 342 and 343, *P* < 0.01) and 9 months (*n*s = 342 and 341, *P* < 0.001). Willett 1979 showed a marginally significantly greater rate of weight gain for children treated with levamisole (2.5 mg/kg, *n* = 166) than those given placebo (*n* = 175) overall [2.08 vs. 1.92 kg/year, percent difference = 8%, *P* = 0.06]. Willett 1979 conducted a sub-analysis of children that had confirmed *Ascaris* infection at baseline (*n* = 97, levamisole = 45, placebo = 52); in the 78 children that were followed for 12 months those given levamisole had significantly greater rate of weight gain than children given placebo [2.31 kg/year vs. 1.91 kg/year, *P* = 0.03]. Donnen 1998 reported that children given mebendazole gained significantly less weight over 12 months [mean weight gain (95% CI): 1.715 (1.474–1.956)] than children given placebo [mean weight gain (95% CI): 2.266 (2.019–2.513)] (*n* = 236, *P* = 0.002). Stephenson 1980 showed greater weight gain in *Ascaris*-infected children who received levimisole (*n* = 61) than non-infected children who received levimisole (*n* = 125) (0.70 kg vs. 0.05 kg, *P* < 0.05), and higher percentage expected weight gain (129.9% vs. 98.2%, *P* < 0.025), calculated as the amount of weight gained divided by the standard expected weight gain, converted to a percentage. None of the other seven articles reported significant differences in measures of weight.

### Women of reproductive age

**Fig. 2:**
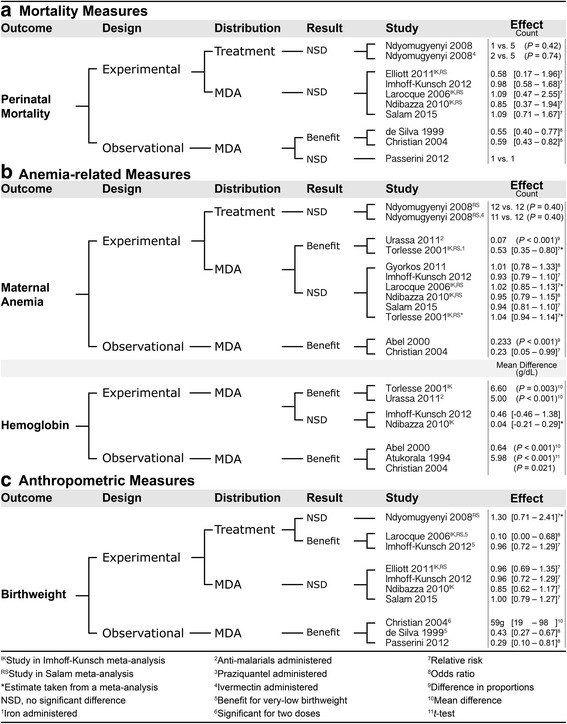
Effects of deworming women of reproductive age. Identified evidence for mortality (panel **a**) anemia-related (panel **b**) and anthropometric measures (panel **c**) is grouped by design, distribution type, and result

We identified 11 studies of women of reproductive age. The studies included five randomized (four individual, one cluster) trials, five repeated cross-sectional studies, and one nonrandomized community-based trial. Eight studies investigated MDA programs, three assessed the effect of deworming on infected children. Additionally, we identified two meta-analyses and one systematic review [38–40] (see Fig. 2). The systematic review (Brooker 2008) was unable to quantify the effects of deworming due to the variety of methods and measures employed, and is not discussed further. We restrict the results reported here to perinatal mortality, maternal anemia, maternal hemoglobin, and birthweight because these measures are of primary importance for our objective and a substantial amount of evidence has been reported. For additional health outcomes see the supplemental material (Additional file 1).

#### Women of reproductive age, perinatal mortality

Two meta-analyses [39, 40] that included three MDA trials [41–43] a single additional treatment trial [44], and three observational MDA studies [45–47] assessed the effect of deworming on perinatal mortality. Christian 2004 reported that infants whose mothers received one dose of albendazole during mid-gestation (12 weeks after urine detection of pregnancy) and one during late-gestation (32 weeks of gestation) (*n* = 2726) were less likely to experience mortality within 6 months than infants of mothers who received zero doses of albendazole (25/261 vs. 116/2981) [relative risk (95% CI:) = 0.59 (0.43–0.82]; there was no significant treatment effect for women who received one dose (88/866). de Silva 1999 found that the proportions of stillbirths and perinatal deaths were lower in infants whose mother recalled having taken mebendazole (*n* = 5275) than in infants whose mother did not recall having taken any anthelminthic (*n* = 1737), among all women who recalled treatment [99/5275 vs. 58/1737, OR (95% CI): 0.55 (0.40–0.77), *P* < 0.001] and among women whose treatment could be confirmed with documentation [62/3540 vs. 56/1670, OR (95% CI): 0.52 (0.36–0.75), *P* < 0.001]. None of the other six studies showed a benefit for deworming on perinatal mortality.

#### Women of reproductive age, anemia-related measures

Two meta-analyses [39, 40] that included one treatment trial [44] and three MDA trials [42, 43, 48], an additional three MDA trials [41–43, 49, 50], and two observational MDA studies [45, 51] assessed the effect of deworming on maternal anemia. Urassa 2011 [50] showed a significantly larger reduction in the prevalence of anemia from first antenatal care visit (12–24 weeks) to term (≥ 36 weeks) in the treatment group (26.1%) than in the placebo group (18.8%) (*P <* 0.001), and there was a significantly greater reduction in the percentage of women who were moderately anemic in the treatment group (30.1%) than the placebo group (21.2%) (*P* < 0.001). Torlesse 2001 reported an increase in the prevalence of anemia between baseline and third trimester in women who received double placebos (*n* = 29, *P* < 0.001), iron supplement and placebo (*n* = 35, *P* = 0.039), or albendazole and placebo (*n* = 29, *P* < 0.001), but not in women who received albendazole and iron (*n* = 32, *P* = 0.61). Abel 2000 reported that the reduction in anemia prevalence was greater in pregnant women who lived in randomly selected intervention districts (receiving 100 mg of albendazole twice per day for three consecutive days and 60 mg of iron supplementation from the fourth month of pregnancy, as well as an intensive information, education, and communication program, in addition to the government prophylaxis program available to all women) than in pregnant women who lived in a district with only the government prophylaxis program available (first trimester difference = 21.7%, second trimester difference = 27.2%, *P <* 0.001, third trimester difference = 23.3%, *P <* 0.001). Christian 2009 reported that women who received albendazole in the second trimester were more likely to be moderately anemic (7–9 g/dL) than severely anemic (< 7 g/dL) in the third trimester (37 moderate vs. 36 severe) than women who did not receive albendazole in the second trimester (one moderate vs. four moderate) after adjustment for possible confounders [adj. OR (95% CI): 0.23 (0.05–0.99)]. The remaining six trials and the two meta-analyses did not find significant differences in rates of maternal anemia.

One meta-analysis [39] that included two MDA trials [43, 48, 50] an additional one MDA trial [50], and three observational studies [45, 51, 52] assessed the effect of deworming on maternal hemoglobin. Torlesse 2001 reported a significantly smaller decline in hemoglobin between the first and third trimester in women who received albendazole than in women who received albendazole placebo [difference = 6.6 g/L, *P =* 0.003] controlling for possible confounders and whether participants received iron supplementation. The difference was 13.7 g/L (*P <* 0.001) for women who received iron-folate supplements rather than iron placebo. Urassa 2011 [50] showed that maternal hemoglobin was significantly higher in the treatment (118 g/L) than placebo (113 g/L) groups at 4 months postpartum (*P <* 0.001). Christian 2004 reported that women who received albendazole in the second trimester (*n* = 829) had increased mean hemoglobin in the third trimester compared to women who did not receive albendazole in their second trimester (*n* = 22) (*P =* 0.021). Abel 2000 reported that pregnant women in the intervention community had higher mean hemoglobin at follow-up than pregnant women in the control community in the first trimester (mean difference = 0.84 g/dl, *P <* 0.01; 95% CI for mean hemoglobin: 11.09 – 11.63 vs. 9.93 – 11.11), second trimester (mean difference = 0.90 g/dl, *P <* 0.001; 95% CI for mean hemoglobin: 10.55 – 10.95 vs. 9.76 – 9.94), and third trimester (mean difference = 0.64 g/dl, *P <* 0.001; 95% CI for mean hemoglobin: 10.25 – 10.73 vs. 9.74 – 9.96). Atukorala 1994 reported that women who received iron supplements and recalled taking an anthelminthic (*n* = 51) had significantly greater mean hemoglobin than women who received iron supplements but did not recall taking an anthelminthic (*n* = 64) [*t* = 5.98, *P* < 0.001]. The other MDA trial and meta-analysis did not show any significant differences.

#### Women of reproductive age, anthropometric measures

Two meta-analyses [39, 40] that included two treatment trials [42, 44], two MDA trials [41, 43], and three observational MDA studies [45–47] assessed the effect of deworming on infant birthweight. Larocque 2006 found a lower proportion of very low birthweight babies in the deworming plus iron group (0 out of 479) than the placebo plus iron group (7 out of 471) [OR (95% CI): 0.1 (0.0–0.68), *P =* 0.007). No significant differences were found in mean birthweight or in the proportion of low birthweight babies (<2500 g). Imhoff-Kunsch 2012 found no significant differences in a meta-analysis with two MDA trials (Elliott 2011 and Larocque 2006) on low birthweight births; however, a significant difference was found in very low birthweight babies in two MDA trials (Larocque 2006 and Ndibazza 2010) [*Z* = 2.16, *P* = 0.03]. Christian 2004 reported that women who received albendazole in their second trimester (*n* = 2726) gave birth to infants with higher mean birthweight than women who received no doses (*n* = 58) after adjustment for possible confounders [mean difference (95% CI): = 59 g; 95% CI: 19 – 98]; there was no significant treatment effect for women who received one dose. de Silva 1999 showed that women who recalled taking mebendazole had significantly fewer very low birthweight babies (<1500 g) than women who did not recall taking any deworming medication overall [59/5271 vs. 40/1735 births, OR (95% CI): 0.47 (0.32–0.71), *P* < 0.001], and the effect was confirmed in women whose mebendazole exposure could be confirmed with medical records [40/3540 vs. 40/1670 births, OR (95% CI): 0.43 (0.27–0.67), *P* < 0.001]. Passerini 2012 showed that women who gave birth in districts that targeted non-pregnant women of reproductive age with four-monthly albendazole and weekly iron and folic acid supplementation had a lower prevalence of low birthweight babies adjusted for clustering and possible confounders (5/168 vs. 22/295 births, adj. OR (95% CI): 0.29 (0.10–0.81), *P =* 0.017), and higher mean birthweight adjusted for clustering [3135 g vs. 3011 g, mean difference (95% CI): 124 g (26–255), *P <* 0.001] than women who gave birth in districts that offered only routine health services. Benefits were not found in the other three trials or the other meta-analysis.

### Meta-analysis of children younger than five

We conducted meta-analyses on studies of children younger than 5 years that reported data for mortality, hemoglobin, height, weight, HAZ, WAZ, WHZ, and/or MUAC. Two studies [18, 26] used a 2 × 2 factorial design with contrasts between groups that received deworming or placebo and groups that received deworming plus a co-intervention (iron supplementation and *Giardia* treatment respectively) or deworming plus placebo, so we included two contrasts for each of these studies. Results for weight are reported below. For analyses of mortality, hemoglobin, height, HAZ, WAZ, WHZ, and MUAC see Additional file 1.

#### Weight

In order to conduct the meta-analyses on deworming on weight in children younger than five, two factors must be investigated: different estimates of effects from studies and inclusion/exclusion of single treatment studies. Disagreements exist about the correct estimates of effect size and uncertainty for two studies relevant to this analysis. Awasthi 2001 reported inconsistent standard errors for weight: one that would suggest significant benefit and another that suggests nonsignificant differences. Taylor-Robinson 2015 appears to use the larger set of standard errors. Croke 2016 provides three arguments for using the smaller standard errors: 1) they contacted the first author, whom they report disagreed with Taylor-Robinson’s standard error estimates; 2) they suggest that the standard error estimate for Awasthi 2001 in Taylor-Robinson 2015 is larger than other trials of similar size included in the meta-analysis; 3) they defer to the authors’ interpretation that the result was significant, which is reported consistently throughout the paper. Welch 2017 writes in the appendix (p. 33) that a request for clarification about this discrepancy was made and received, but does not state which estimate was used in their analysis. Sur 2005 provides weight data graphically, but not numerically. Taylor-Robinson appears to use differences at follow-up, whereas Croke 2016 extracted difference-in-difference estimates from baseline to follow-up using an online tool, which reduces the standard error estimate and makes the finding significant. Welch 2017 does not clarify which estimate was used in their analyses.

Taylor-Robinson 2015 also differs from Croke 2016 and Welch 2017 on inclusion of weight estimates from two studies relevant to this analysis. Taylor-Robinson excludes Gupta 1982 citing the randomization method, whereas Croke 2016 and Welch 2017 include this estimate. Taylor-Robinson 2015 does not include Willett 1979, but lists their estimates in a section detailing trials that provided data in a format that could not be used, whereas Croke 2016 and Welch 2017 include their estimate.

Another area of discussion has been differences in the effects of deworming between single dose and multiple doses. Some studies have found that single dose deworming treatments can be less effective for some helminth species [53, 54]. Given these two factors we ran four primary meta-analyses on the impact of deworming on weight in children younger than 5 years old.

**Fig. 3:**
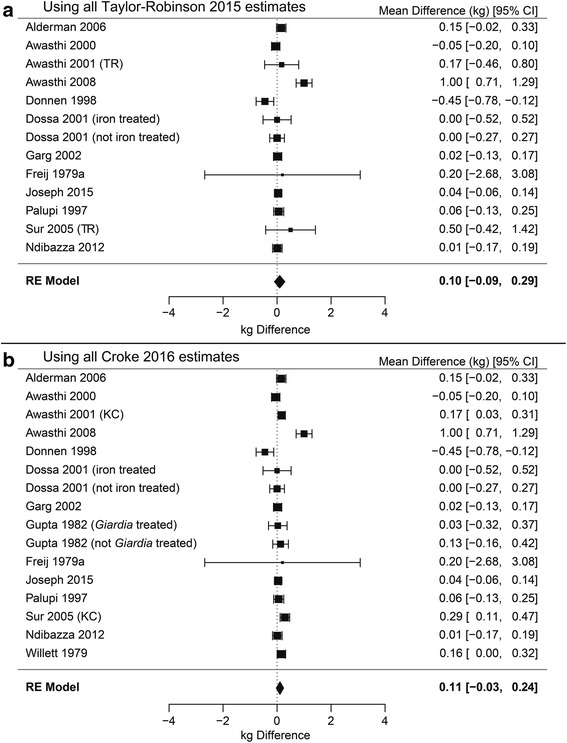
Forest plots of pooled difference in weight (kg) including all single- and multiple-dose studies. Meta-analyses with single-dose studies included using the estimates used in Taylor-Robinson’s 2015 Cochrane review (**a**) and estimates used by Croke 2016 (**b**). Neither meta-analysis is significant, *P* = 0.322 and *P* = 0.117, respectively

**Fig. 4:**
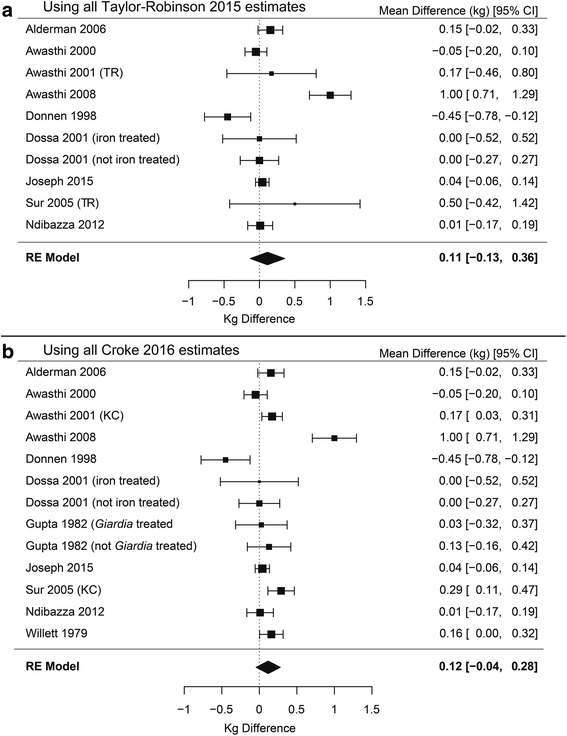
Forest plots of pooled difference in weight (kg) including only multiple-dose studies. Meta-analyses with only multiple-dose studies included using the estimates used in Taylor-Robinson’s 2015 Cochrane review (**a**) and estimates used by Croke 2016 (**b**). Neither meta-analysis is significant, *P* = 0.359 and *P* = 0.148, respectively

The results of the four meta-analyses are shown in Figs. 3 and 4 on weight in children younger than 5 years from 14 studies (11 multiple-dose, three single-dose) [17–19, 25–28, 30–35]. Figure 3 shows the meta-analyses when single-dose studies are included with 3a using the estimates given by Taylor-Robinson 2015 and 3b the results using estimates given by Croke 2016. In neither case is there a significant effect of deworming on weight. Figure 4 presents the same two meta-analyses, this time restricted to studies that have multiple doses. Again, neither the meta-analysis using the estimates from Taylor-Robinson 2015 (3a) or Croke 2016 (3b) found a significant effect of deworming on weight (see Fig. 4).

### Robustness of effects

**Fig. 5:**
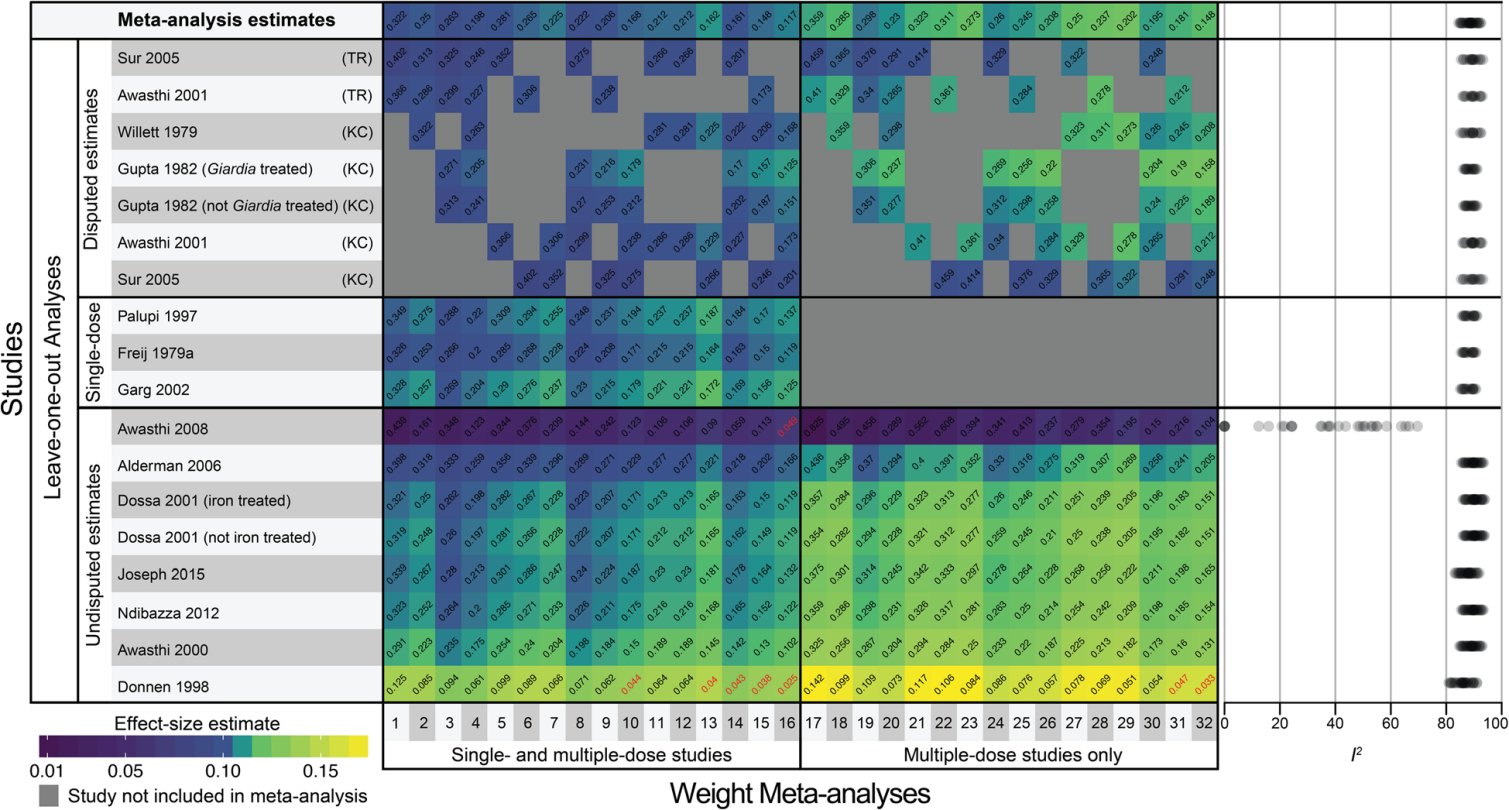
Heat map of robustness checks. Robustness checks of weight meta-analyses for children younger than five. Effect-size estimates for all of the 32 main meta-analyses are shown along the top row, with estimates from leave-one-out analyses are below. Effect size is indicated by color warmth (warmer colors are larger effect sizes) with *P*-values written diagonally on each estimate. *P*-values below 0.05 are shown in red. Heterogeneity, as indicated by *I* ^*2*^, is plotted from each of the 32 meta-analyses to the right of the heat map. Effect sizes became significant in 8 out of 450 estimates. Heterogeneity is above 80% in all robustness checks, except when Awasthi 2008 is dropped, in which case it is below 80% for all 32 models

In order to check the robustness of these results, we systematically varied the inclusion of estimates from Taylor-Robinson 2015 and Croke 2016 (see Fig. 5). We conducted a meta-analysis on every combination of the four disputed estimates, resulting in 16 meta-analyses (four studies with two estimates each means there were 4^2^ possible combinations). We then repeated these analyses excluding single-dose studies, resulting in a total of 32 meta-analysis models. The effect sizes and *P*-values are indicated along the top row of Fig. 5. The first column of Fig. 5, (meta-analysis 1) corresponds the meta-analysis including all Taylor-Robinson 2015 estimates (Fig. 3a), column 16 corresponds to the meta-analysis including all estimates from Croke 2016 (Fig. 3b). Column 17 and 32 correspond to the meta-analyses using Taylor-Robinson 2015 and Croke 2016 estimates, respectively, excluding single-dose studies (Fig. 4a and b).

None of the 32 meta-analyses were significant at the 95% or 90% confidence levels. The effect size estimates from the 32 meta-analyses ranged from. 0.09 to 0.11 kg when single-dose trials were included (*P*-values from 0.117 to 0.322), and from 0.10 to 0.12 when single-dose studies were excluded (*P*-values from 0.148 to 0.359). The smallest observed *P*-value (*P* = 0.117) was obtained using all of the estimates suggested by Croke with single-dose studies included. Heterogeneity was substantial in all models, with *I* ^*2*^ ranging from 84.43% to 89.91% when single-dose trials were included, and from 86.33% to 92.54% when they were excluded.

In addition to the initial robustness checks, we performed leave-one-out analyses for all studies in each of the 32 meta-analyses to investigate whether the effect in in any model was being driven by any single study. These leave-one-out analyses in combination with the main 32 meta-analysis models resulted in 450 estimates of the effect of deworming on weight. Leave-one-out analyses showed that results were robust to dropping all estimates except for Donnen 1998 and Awasthi 2008 in eight cases (*P*-values shown in red in Fig. 5). Heterogeneity was above 80% in all of the meta-analyses except those where Awasthi 2008 was dropped.

## Conclusions

The primary objective of this paper was to investigate the evidence for benefits of deworming in order to decide whether it should be included as an intervention in *LiST*. The evidence suggests that deworming should not be included for any health outcome in children younger than five or women of reproductive age. A substantial amount of evidence in children younger than 5 years suggests that there is no population effect on any health outcome. We investigated the only health outcome where there is even a suggestion of a benefit (weight) using an approach that should have allowed us to reveal a benefit if one existed. It is notable that our robustness checks revealed a significant effect in a number of analyses well below what would be expected simply from type I error. There have been fewer studies of pregnant women, but the balance of the evidence points to no benefit of deworming in either population.

There are many sources of heterogeneity in studies of deworming, including differences in types and mix of infection (hookworm, *Ascaris*, *Trichuris*) and co-infection (e.g., *Schistosomiasis*, *Giardia*, *Plasmodium*), deworming treatments administered (e.g., albendazole, mebendazole, piperazine) and co-interventions (e.g., iron supplementation, praziquantel, sulfadoxine pyrimethamine), location, and length of follow-up. The short follow-up (e.g., one-month) in some studies is of particular concern as it is unlikely that measures such as child growth will change drastically in such a short interval. However, in looking across studies and using multiple approaches to search for significant population impact on health, such as excluding single-dose studies, we find no evidence of any population-level effects.

A key barrier to understanding the effect of deworming is the variety of prevalence and intensity levels in treated populations. A number of studies investigated the effects of deworming in areas below the 20% prevalence threshold for WHO mass treatment recommendations. Additionally, some researchers suggest that the majority of morbidity attributable to STH is due to high-intensity infections in a small proportion of the infected population, further hampering detection of a treatment benefit and causing some to call the Cochrane methodology inappropriate for this intervention [10, 12].

Ideally, researchers would have complete knowledge of infection prevalence and intensity and nutritional status for a large study population with heterogeneous infections, and without confounding factors [54]. In such conditions one would expect an effect at the population level. It is notable that studies that have conducted sub-analyses of infected individuals (e.g., Joseph 2015, Ndyomugyenyi 2008) have not consistently showed a benefit for deworming. However, even in a study where all of these conditions are not met, simple statistical techniques should allow researchers to investigate whether there is an effect on a subset of the population. For example, researchers have suggested that iron deficiency anemia is linearly related to hookworm intensity above a threshold of 2000 epg [52, 55]. If a treatment effect exists, studies that have effectively treated hookworm and Trichuris should show a bimodal distribution in post-treatment hemoglobin levels due to a dose-response. That is, uninfected and lightly infected individuals should be randomly distributed around zero change in hemoglobin, while moderately to heavily infected individuals should show a substantial increase in hemoglobin levels. In addition to basic exploratory data analysis, more sophisticated techniques such as k-nearest neighbor analysis, k-means analysis, and finite mixture models may be able to shed light on any purported relationship [56]. The dynamic nature of the interactions between human health and STHs makes revealing a benefit of deworming treatments on measurable outcomes difficult, but it is incumbent upon researchers to provide evidence to support this intervention.

The epidemiology of STH varies by species and geographic region, but children younger than five do not tend to suffer the largest burden of STH. Children as young as 6 months can be infected with STH [55], but evidence suggests that *Ascaris* and *Trichuris* reach peak prevalence at four and 7 years respectively, and hookworm tends to peak in adolescence [12, 56]. *Ascaris* and *Trichuris* age-intensity profiles have convex shapes with a peak around 7 years, whereas hookworm intensity generally increase monotonically until 15 to 25 years and then stabilize [12, 56]. Due to the nonlinear relationship between infection prevalence and intensity [57], these age-prevalence and age-intensity profiles suggest that that older individuals are more likely to suffer morbidity due to STH and thus show benefits of deworming.

The evidence for effects of deworming in pregnant women is limited, in part because the practice was not recommended prior to a 1999 study that found no adverse birth outcomes [46]. Some studies have identified benefits for perinatal mortality, maternal anemia, hemoglobin, iron deficiency, and birthweight. A limited number of studies have found benefits of deworming women of reproductive age in perinatal mortality, maternal anemia, hemoglobin, and birthweight. However, RCTs and meta-analyses have not confirmed these findings, and these studies have suffered from a variety of limitations, including small sample sizes, confounding by co-interventions, loss to follow-up, and observational design.

This review considers the narrow question of whether to include deworming as a population health intervention for children younger than five and women of reproductive age in the *LiST* software. Given the scope of available evidence for benefits of deworming on child and maternal health, we do not currently recommend including deworming in the *LiST* software. While the current evidence does not support including the effect of deworming on population health in the *LiST* software, this does not mean that there are no beneficial effects of deworming. It is possible that the benefits of deworming in research to date have been diluted. In addition, not all studies report prevalence and intensity data, and the complex interaction between these factors and morbidity makes post-hoc adjustment difficult. Researchers have suggested that deworming individuals is inherently good [10]. We do not disagree with this assertion, but there does not appear to be a measurable benefit at the population level in either children under five or pregnant women.

## Additional file

Additional file 1: Additional evidence. Analyses and study characteristics that were not included in the main article for the purpose of brevity are included in this file. (DOCX 14103 kb)

## Abbreviations

HAZ: Height-for-age *Z*-score
*LiST*: Lives Saved Tool
MDA: Mass drug administration
MUAC: Mid-upper arm circumference
STH: Soil-transmitted helminth(s)
WAZ: Weight-for-age *Z*-score
WHZ: Weight-for-height *Z*-score
