# Supplementary material for: Effects of deworming on child and maternal health: a literature review and meta-analysis
Source: BMC Public Health. 2017 Nov 7;17(Suppl 4):830. doi: 10.1186/s12889-017-4747-0 (PMC5688423; doi:10.1186/s12889-017-4747-0)
Supplement: Additional file 1: — Additional evidence. Analyses and study characteristics that were not included in the main article for the purpose of brevity are included in this file. (DOCX 14103 kb) [file 12889_2017_4747_MOESM1_ESM.docx]

**Additional File 1**

***Children younger than five years***

Supplementary Table 1. Characteristics of studies in children younger than five.

| Study | Age (months) | Drug type | Dosage  (mg) | Follow-up (months) | Prevalence  (%) |
| --- | --- | --- | --- | --- | --- |
| Alderman 2006 | 12 – 84 | albendazole | 400 | 36 | > 50 |
| Awasthi 2000 | 18 – 42 | albendazole | 600 | 24 | < 20 |
| Awasthi 2001 | 6 – 18 | albendazole | 400 | 18 | < 10 |
| Awasthi 2008 | 12 – 60 | albendazole | 400 | 24 | ≤ 20 |
| Awasthi 2013 | 6 – 72 | albendazole | 400 | 60 | > 20 |
| Bhutta 2009 | 6 – 24 | mebendazole | 600 | 3 | < 20 |
| Donnen 1998 | 0 – 72 | mebendazole | 500 | 12 | < 20 |
| Dossa 2001 | 36 – 60 | albendazole | 600 | 10 | > 50 |
| Freij 1979 | 18 – 60 | piperazine | 3000 | 1.06 | 100 |
| Freij 1979b | 12 – 48 | piperazine | 3000 | 1.06 | ~ 50 |
| Garg 2002 | 24 – 48 | mebendazole | 500 | 6 | > 50 |
| Goto 2009 | 3 – 15 | albendazole | 200 (ml) | 3 | < 10 |
| Greenberg 1981 | 18 – 96 | piperazine | 80 (per kg) | 11 | > 50 |
| Gupta 1982 | 24 – 61 | piperazine | 75 (per kg) | 12 | > 50 |
| Jalal 1998 | 36 – 72 | levamisole | not reported | 1 | 100 |
| Joseph 2015A | 12 – 24 | mebendazole | 500 | 12 | < 20 |
| Joseph 2015B | 15 – 60 | mebendazole | 500 | 12 | < 20 |
| Kirwan 2010 | 12 – 59 | albendazole | 400 | 14 | ≥ 50 |
| Kloetzel 1982 | 12 – 96 | mebendazole | 100 | 10 | > 50 |
| Ndibazza 2012 | 15 – 60 | albendazole | 400 | 60 | < 10 |
| Northrop 1987 | 22 – 108 | pyrantel paomoate | 10 (per kg) | 0.33 | 100 |
| Northrop-Clewes 2001 | 24 – 60 | mebendazole | 500 | 12 | > 50 |
| Palupi 1997 | 24 – 60 | albendazole | 400 | 2.5 | > 50 |
| Reddy 1986 | 12 – 60 | tetrimisole | 50 | 12 | > 20 |
| Rousham 1994 | 12 – 72 | mebendazole | 500 | 18 | > 50 |
| Stephenson 1980 | 12 – 72 | levamisole | 40–80 | 0.47 | 100 |
| Stoltzfus 2001 | 6 – 59 | mebendazole | 500 | 12 | > 20 |
| Stoltzfus 2004 | 6 – 71 | mebendazole | 500 | 12 | > 50 |
| Sur 2005 | 12 – 60 | albendazole | 400 | 12 | > 50 |
| Tanumihardjo 1996 | 8 – 80 | albendazole | 400 | 1 | 100 |
| Tanumihardjo 2004 | 44.4 ± 14.4 | albendazole | 400 | 1 | 100 |
| Willett 1979 | 6 – 91 | levamisole | 2.5 (per kg) | 12 | > 20 |

The following are additional outcomes that we investigated in children younger than five years. See Supplementary Figure 1.

**Supplementary Figure 1. Evidence for an effect of deworming in children younger than five years**

**

**

*Mortality.* Six MDA trials [1-6] reported the effect of deworming on mortality in children younger than five. None of the trials showed a significant difference in mortality between children who received deworming medications compared to children that did not.

*Anemia-related measures* Three MDA trials [7-9] assessed the impact of deworming on anemia prevalence. Palupi 1997 demonstrated a significant reduction in the prevalence of anemia in the iron only (*n* = 96, *P <* 0.01) and iron plus deworming (*n* = 95, *P <* 0.001) groups, but not in the placebo group (*n* = 98, *P =* 0.17). Anemia prevalence was significantly higher in the placebo group than in the combined iron-treated groups at the end of the study (*P =* 0.04). Stoltzfus 2004 did not show a benefit for deworming on prevalence of anemia overall, however a post-hoc analysis revealed that children less than 24 months old had a significant reduction in moderate anemia [adjusted odds ratio (adj. OR) (95% CI): 0.41 (0.18–0.94)].

One MDA trial [10] evaluated the effect of deworming on serum ferritin and erythrocyte protoporphyrin concentration. Dewormed children had significantly lower serum ferritin (47.9 vs. 58.1 µg/l, *P* = 0.037) at 12-month follow-up. One study (Stoltzfus 2001) measured erythrocyte protoporphyrin concentration; no significant differences were found. One study (Northrop-Clewes 2001) [11] assessed the effect of deworming on plasma albumin, but did not find any significant differences.

Two MDA trials [12, 13] and one observational treatment study [14] investigated the impact of deworming on plasma albumin. Northrop 1987 did not find any significant differences overall, however a subgroup of children infected with *Ascaris* (*n* = 56) had lower baseline plasma albumin levels than uninfected children (*n* = 38) [mean (SEM): 35.72 (1.32) vs. 39.62 (0.91), ANOVA regression *p* < 0.01], and after treatment plasma albumin levels of successfully treated children increased significantly [*n* = 56, Mean (SEM) pre-treatment = 35.72 (1.32) vs. post-treatment = 38.07 (1.34), paired *t*-test *p* < 0.005], and were no longer significantly different than uninfected children.

*Anthropometric measures* Three MDA trials [1, 10] assessed the impact of deworming on the number of children stunted (HAZ < -2) and wasted (WAZ < -2), and one trial assessed the effect of deworming on the number of underweight children (WHZ < -2). Awasthi 2000 found that there was a significantly smaller increase in the number of children stunted in the albendazole than the placebo group [increase (95% CI): 2.06% vs. 11.44% (6.01–12.75), *P* < 0.001]. These three trials found no other significant differences in these counts.

Ten MDA trials [1-3, 5, 7, 11, 15-18] assessed the effect of deworming on height; two of these trials (Garg 2002 and Joseph 2015) conducted a sub-group analysis with only helminth-infected individuals. One study (Awasthi 2013) [4] reported observational results for height. None of the studies showed a significant difference in measurements of height between groups overall. Garg 2002 found no significant height differences overall, however in a sub-analysis of helminth-infected children, those who received mebendazole (*n* = 22) had significantly greater height gains after six months than those who received placebo (*n* = 20) [mean (SE): 4.44 (0.26) cm vs. 3.66 (0.20) cm, *P <* 0.04]. Joseph 2015 found no significant differences overall, however in a subgroup analysis of STH infected children who received mebendazole or placebo at either 12 months, 18 months, both, or neither (*n*s: mebendazole/placebo = 50, placebo/mebendazole = 48, mebendazole/mebendazole = 56, placebo/placebo = 32), those who received mebendazole at 12 months and placebo at 18 had marginally significantly less height (length) gain before adjustment for possible confounders [mean difference (95% CI): -0.68 (-1.42–0.05), *p* = 0.068] and after adjustment [mean difference (95% CI): -0.68 (-1.42–0.07), *p* = 0.077].

Eleven MDA trials [1, 2, 6-9, 11, 12, 15, 16, 19] investigated height-for-age; one of these trials [15, 18] conducted sub-group analyses with only helminth-infected individuals. Awasthi 2000 reported a significantly smaller increase in stunting (HAZ < -2.0) in children who received albendazole than children who received placebo (reported previously). Garg 2002 found no significant differences in height-for-age overall, however their sub-group analysis of helminth-infected children revealed marginally significantly greater height-for-age gains in treated (*n* = 22) than untreated (*n* = 20) children [mean (SE): 0.00 (0.08) vs. 0.22 (0.09), *P =* 0.07]. The other nine trials showed no significant benefits for deworming.

Nine MDA trials [1, 6, 12-16, 18-20] assessed the effect of deworming on weight-for-age; two of these conducted sub-analyses on helminth infected children. None of the studies showed a benefit of deworming overall. Rousham 1994 reported that children randomized to mebendazole (*n* = 688) had significantly smaller increases in unadjusted analyses of WAZ than children randomized to placebo (*n* = 714) [*Z* = 0.03 vs. 0.12, *F* = 4.33, *P* < 0.05], however the groups were not significantly different after adjustment for possible confounders. Garg 2002 found no significant differences in weight-for-age overall, however their sub-group analysis of helminth-infected children revealed greater weight-for-age gains in treated (*n* = 22) than untreated children (*n* = 20) (0.13 ± 0.08 vs. 0.41 ± 0.08, *P =* 0.02). Joseph 2015 showed no differences overall or in sub-analyses. The other six studies found no significant differences.

Eight MDA trials [6-8, 11-13, 15, 19, 20] investigated the effect of deworming on weight-for-height; one of these trials (Garg 2002) conducted a sub-group analysis with only helminth-infected individuals. None of the studies showed a significant benefit of deworming overall or in subgroup analyses. Stoltzfus 2004 also showed a significant benefit for deworming on mild wasting (WAZ < -1.0) in children younger than 30 months after adjusting for age, sex, mid-upper arm circumference, and stunting (adj. OR = 0.38; 95% CI: 0.16 – 0.90). Dossa 2001 showed no significant difference between iron only, iron plus deworming, deworming only, and placebo groups overall or in subgroup analyses. Rousham 1994 reported that children randomized to mebendazole (*n* = 688) had significantly larger decreases in unadjusted analyses of WHZ than children randomized to placebo (*n* = 714) [*Z* = -0.25 vs. -0.05, *F* = 22.28, *P* < 0.001]. Children that received six or more doses had the largest decreases [*F* = 9.51, *P* < 0.001], however decreases in WHZ were not significantly different after adjustment for possible confounders. The other five studies did not show any significant differences.

Two quasi-randomized treatment trials [21], four MDA trials [5, 7, 9, 11, 13], and one observational study [22] evaluated the effect of deworming on mid-upper arm circumference (MUAC). None of the studies showed a benefit of deworming overall. Stoltzfus 2004 showed a significant benefit of deworming for children younger than 30 months, but not older than 30 months, after adjusting for age, sex, weight-for-height circumference, and stunting [adj. OR (95% CI): 0.29 (0.09–0.91)].

One study [4] reported observational results of the effect of deworming on body mass index (BMI); no significant differences were found between treated and untreated groups.

Two quasi-randomized treatment trials (Freij 1979a and Freij 1979b) [21] and one observational study [22] investigated the effect of deworming on triceps skinfold thickness. Stephenson 1980 showed that children infected with *Ascaris* had a 1.87mm greater mean decrease in triceps skinfold thickness [*ß* = -1.84, SE = 0.13, *F* =183.83, *P <* 0.001] in the 14 weeks before deworming than children who were not infected, after controlling for a variety of demographic, parasitic, clinical, public health, and socioeconomic factors. In the 14 weeks after deworming, *Ascaris* infected children treated with levimisole had 3.11mm greater increase in triceps skinfold thickness than uninfected children treated with levimisole [SE = 0.18, *F* = 297.29, *P* < 0.001], controlling for other factors. Neither trial from Freij 1979 found a significant difference in triceps skinfold thickness between children who received deworming medication compared to those who did not.

*Other measures.* One quasi-randomized treatment trial [21] investigated the impact of deworming on blood xylose, fat excretion, and nutrient excretion. No significant benefit of deworming on these metrics was found.

Two treatment trials [23, 24] and one MDA trial [25] examined the effect of deworming on serum retinol. Jalal 1998 found that *Ascaris* infected children with high egg counts (>3200 eggs per gram [epg]) who received a combination of a basic meal, added dietary fat, and levimisole had increased serum retinol compared to children who received only a basic meal (interaction effect: *P <* 0.05). In a second sub-study, *Ascaris* infected children with high egg counts (>3200 epg) who received a basic meal, *ß*-carotene-rich food, and levimisole had increased serum retinol compared to children who received a basic meal and *ß*-carotene-rich food alone (interaction effect: *P <* 0.001). The other two studies showed no significant benefits for deworming.

One MDA trial [6] assessed the impact of deworming on cytokine response and immunoglobulin levels; no significant differences were found.

One MDA trial [11] investigated the effect of deworming on α_1_-antichymotrypsin and intestinal permeability; no significant differences were found.

Two MDA trials [12, 13] and one observational study [14] assessed the effect of deworming on intestinal permeability. Goto 2009 found that 36 weeks into secnidizole treatment for *Giardia* (75 mg/ml) and albendazole treatment for STH, children that received full doses of both deworming medications (*n* = 75) had significantly lower lactulose/mannitol ratio (L:M/ratio) than children who received placebos (*n* = 88) [geometric mean L:M/ratio (SD): Both full dose = 0.14 (0.33), both placebos [FC] = 0.18 (0.33), *P* = 0.034]. The other MDA trial and observational study did not find any significant benefits of deworming.

Three MDA trials [2, 6, 26] assessed the effect of deworming on infectious disease incidence and immune responses and one study reported observational results (Tanumihardjo 2004) [4]. Awasthi 2000 reported no significant difference in incidence of measles. Kirwan 2010 demonstrated a significantly slower increase in *Plasmodium* infection for the placebo than the albendazole group (proportional change in odds = 0.94, *P =* .002). Ndibazza 2012 reported that quarterly deworming during the first five years of life had no significant impact on cytokine responses to antigen 85, tetanus toxoid, or tetanus toxoid immunoglobulin, however deworming appeared to decrease interferon-*γ* (INF-*γ*) response to crude filtrate proteins of *Mycobacterium tuberculosis* (geometric mean ratio = 0.73 pg/ml; 95% CI: 0.56 – 0.96) as well as interleukin-13 (IL-13) response (0.71 pg/ml; 95% CI: 0.55 – 0.94), contrary to the authors’ predictions. Awasthi 2013 showed no significant differences in illness in the past four weeks for children in deworming districts than for those in control districts.

***School-aged children***

We identified 22 studies of school-aged children. The studies included 19 randomized (15 individual, 5 cluster) trials, one cross sectional study, and one study that presented cross sectional as well as matched data. Sixteen studies investigated MDA programs and six studies assessed the effect of deworming on infected children. Additionally, we identified two meta-analyses (Taylor-Robinson 2015 and Croke 2016) [27, 28], and two articles (Aiken 2015 and Davey 2015) [29, 30] that reanalyzed data from a previously published study. We report the meta-analyses are with results for school-aged children because they included studies of children up to 16 years old (see Supplementary Figure 2).

**Supplementary Figure 2. Evidence for an effect of deworming in school-aged children**

**



**

*Mortality* None of the identified studies investigated mortality in school-aged children.

*Anemia-related measures* Eight MDA trials [31-39], one treatment trial [31], one re-analysis [29], and one observational study [40] assessed the effect of deworming on anemia. Bhargava 2003 showed that children with heavy hookworm infections (> 400 epg) treated with albendazole and/or heavy *Schisomiasis haematobium* infestations (>50 eggs per 10ml urine) treated with praziquantel had a significant reduction in anemia prevalence from baseline (*n* = 284) to follow-up (*n* = 266) at cutoffs of 1.2 g/dL (67% to 44%), 1.1 g/dL (36% to 14%), and 1.0 g/dL (11% to 3%), but these changes were not compared to the uninfected control group. Miguel 2004 labels the reduction in the proportion of anemia between treated (*n* = 292) and untreated (*n* = 486) children significant (*P* < 0.05), however Aiken 2015 found that this was erroneous (*P* = 0.194). Rohner 2010 demonstrated a significant reduction in the risk of anemia for children who were treated with albendazole and praziquantel (OR = 0.41, *P <* 0.01; 95% CI: 0.3 – 0.7), but no significant reduction in children who received iron fortified biscuits or intermittent preventative malaria treatment. Sufiyan 2011 showed that children in a village that received albendazole and weekly hygiene education had decreased prevalence of anemia (χ^2^ = 29.2, *df* = 1, *P <* 0.001), while those from a comparison village that did not receive the interventions showed no significant reduction.

One MDA trial [37] assessed the effect of deworming on iron deficiency; no significant differences were found.

One treatment trial [41], nine MDA trials [34-39, 42, 43], one meta-analysis [28], and two cross-sectional studies [40, 44] assessed the impact of deworming on hemoglobin levels. Bhargava 2003 reported a significant increase in hemoglobin after 15 months for heavily hookworm-infected children who were treated with albendazole (*n* = 56, *P <* 0.001) but not in non-infected children (*n* = 116) given the same treatment. Friis 2003 showed a significantly greater increase in mean hemoglobin after eight months for children treated with albendazole (*n* = 279) than for children treated with placebo (*n* = 296), adjusted for baseline hemoglobin, age, sex, and height-for-age *Z*-score (*ß* = 2.0, *P =* 0.03; 95% CI: 0.2 – 3.9). Taylor 2001 demonstrated that children who received either iron supplementation or placebo with praziquantel and albendazole (*n* = 236) for three consecutive days maintained their hemoglobin level after six months, whereas children who received either iron supplementation or placebo with anthelminthic placebos (*n* = 192) had a significant mean decrease in hemoglobin after six months (*P <* 0.05). Children who received iron supplementation with praziquantel and three doses of albendazole (*n* = 63) had a significant increase in hemoglobin after 12 months (mean difference = 3.5 g/L; 95% CI: 0.5 – 6.5, *P =* 0.02), whereas children who received placebo with praziquantel and three doses of albendazole had a significant decrease (mean difference = -3.8 g/L; 95% CI: -6.9 – -0.7, *P =* 0.02). Rohner 2010 found that children treated with albendazole and praziquantel had a modest but significant increase in mean hemoglobin concentration (2.4 g/L, *P <* 0.01; 95% CI: 1.2 – 3.7), while children treated with iron fortification and children treated prophylactically for malaria with sulfadoxine pyrimethamine showed no significant difference. Sufiyan 2011 reported that children in a village who received a single dose of albendazole and weekly hygiene lectures for three months had a significant mean increase in hemoglobin in a measurement taken one month later (10.4 g/L vs. 12.4 g/L, paired-*t* = 13.96, *df* = 298, *P <* 0.001), while children in a village that did not receive these interventions also had a smaller, though also significant, increase (10.5 g/L vs. 11.2 g/L, paired-*t* = 2.89, *df* = 298, *P =* 0.004). The other eight studies found no significant differences.

One MDA trial [34] reported the impact of deworming on hematocrit, albumin, and serum iron; no significant differences were found.

Four MDA trials [31, 34-36] assessed the effect of deworming on serum ferritin. Bhargava 2003 reported a significant increase in serum ferritin after treatment with albendazole for hookworm-infected children (*F*(1,326) = 4.0, *P =* 0.019), but no significant difference for non-infected children. Other studies found no significant differences.

One cross-sectional treatment study [44] assessed the effect of deworming on plasma iron. Karyadi 1996 found that, of children infected with either *Ascaris lumbricoides* or *Trichuris trichuira*, those given albendazole (*n* = 53) had a significantly greater increase in plasma iron 10 days after intervention than those given placebo (*n* = 53) (*P =* 0.012).

One MDA trial [37] investigated the impact of deworming on plasma ferritin; no significant differences were found.

*Anthropometric measures* Three treatment trials [41, 45, 46], five MDA trials [34, 47-50], one meta-analysis [28], and one cross-sectional treatment study [44] assessed the impact of deworming on height; none found a significant benefit. Tee 2013 found that at six months children treated with albendazole (*n* = 18) showed less height gain than children treated with placebo (*n* = 19) [3.5 vs. 4.1 cm, *P* = 0.04], but these differences were not significant at 12 months.

Three treatment trials [41, 45, 46], five MDA trials [34, 47-50], two meta-analyses [27, 28], and one cross-sectional treatment study [44] assessed the impact of deworming on weight. Adams 1994 found that children given 400mg of albendazole on three consecutive days (*n* = 28) had significantly greater mean weight gain nine weeks later than children given placebos (*n* = 27) [1.0 ± 0.60 vs. 0.3 kg ± 0.10, difference = 0.7, one-tailed *P* < 0.001]. Adams 1994 also showed a significant increase in weight for unit reduction in hookworm epg [*ß* = -0.193, SE = 0.031, *t* = -6.20, *P* < 0.001]. Stephenson 1993 found that children who were randomized to receive either 600mg of albendazole at each of two visits in a six month period (*n* = 95) or one dose of albendazole and one dose of placebo (*n* = 96) had significantly greater mean weight gain at eight-month follow-up than children who received two doses of placebo (*n* = 93) [zero albendazole = 2.2kg ± 0.12, one albendazole 3.3kg ± 0.18, two albendazole 3.1g ± 0.14, ANOVA *P* Tukey Honestly Significant Difference (HSD) < 0.001 for zero albendazole]. In a meta-analysis of five trials conducted in samples that were either all infected with STH or had high prevalence (Freij 1979a, Stephenson 1989, Stephenson 1993, Sarkar 2002, and Yap 2004), Taylor-Robinson 2015 a significant benefit in weight for children who received a single-dose deworming treatment (*n* = 319) compared to children in control groups (*n* = 308) [mean difference (95% CI): 0.75 (0.24–1.26)]. Taylor-Robinson 2015 found no significant differences in a meta-analysis with 10 MDA trials. Croke 2016 conducted a meta-analysis of twenty MDA trials that provided multiple doses of deworming medications. Croke 2016 found that children who received deworming had greater weight gain than children in control groups in areas of any STH prevalence [mean difference (95% CI): 0.134 kg (0.031–0.236), *P* = 0.01], areas with greater than 20% prevalence [0.148 kg (0.039–0.258), *P* = 0.008], and in areas with greater than 50% prevalence [0.182 kg (0.070–0.293), *P* = 0.001]. The other seven studies found no significant benefits for deworming.

One treatment trial [41], five MDA trials [34, 38, 39, 48, 49], one re-analysis [30], and one cross-sectional treatment study [44] investigated the impact of deworming on height-for-age. Miguel 2004 [39] found that children in the treatment group had marginally significantly greater HAZ than children in the control group [-1.13 vs. -1.22, *P* = 0.09]. The other six studies found no significant benefit for deworming.

One treatment trial [41], five MDA trials [34, 39, 48, 49], one re-analysis [30] and one cross-sectional treatment study [44] assessed the impact of deworming on weight-for-age. Adams 1994 found that children infected with hookworm, *Trichuris trichiura*, and/or *Ascaris lumbricoides* that were treated with 400mg of albendazole for three consecutive days had significantly greater increase in weight *Z*-scores than children given placebo [mean *Z* (SE): 0.30 (0.024) vs. 0.08 (0.034), difference = 0.22, *P* < 0.001]. Stephenson 1993 found that children who received two doses of albendazole or one dose each of albendazole and placebo had significantly greater change in percent weight-for-age than children who received two doses of placebo (zero albendazole = -1.4% ± 0.28, one albendazole = 1.9% ± 0.36, two albendazole = 1.3% ± 0.30 Tukey HSD < 0.0001 for zero doses). The other six studies did not show a significant benefit of deworming.

Two treatment trials [41, 45], two MDA trials [48, 49], and one cross-sectional treatment study [44] investigated the effect of deworming on weight-for-height. Adams 1994 found that children infected with hookworm, *Trichuris trichiura*, and/or *Ascaris lumbricoides* that were treated with 400mg of albendazole for three consecutive days had significantly greater increase in weight-for-height *Z*-scores than children given placebos [mean *Z* (SE): -0.33 (0.036) vs. -0.29 (0.060), difference = 0.30, one-tailed *P* < 0.001]. Stephenson 1993 reported that children given two or one albendazole had greater change in percent weight-for-height than children who received no albendazole [mean percent change (SE) zero albendazole = -0.3% (0.30), one albendazole = 2.8% (0.36), two albendazole = 2.6% (0.30), Tukey HSD < 0.0001 for zero doses]. The other three studies showed no effect of deworming.

One treatment trial [41], one MDA trial [49], and one meta-analysis [28] investigated the impact of deworming on MUAC, triceps skinfold thickness, and subscapular skinfold thickness. Adams 1994 reported that children who received deworming treatments showed significantly greater increases in mid-upper arm circumference than children who received placebo (0.6cm ± 0.07 vs. 0.3cm ± 0.05, difference = 0.3cm, *P =* 0.0002), triceps skinfold thickness (1.0mm ± 0.13 vs. 0.2mm ± 0.09, difference = 0.8mm, *P =* 0.0002), and subscapular skinfold thickness (0.9mm ± 0.10 vs. 0.1mm ± 0.18, difference = 0.8mm, *P =* 0.0002). Stephenson 1993 also reported that children who received one or two deworming treatments had significantly greater mean increases than children who received only placebos in mid-upper arm circumference (zero albendazole = 0.3cm ± 0.40, one albendazole = 0.8cm ± 0.05 two albendazole = 0.7cm ± 0.05, Tukey HSD < 0.0001 for zero albendazole group), triceps skinfold thickness (zero albendazole = 0.2mm ± 0.08, one albendazole = 2.0mm ± 0.11, two albendazole = 2.0mm ± 0.12, Tukey HSD < 0.0001 for zero albendazole group), and subscapular skinfold thickness (zero albendazole = 0.40mm ± 0.08, one albendazole = 1.8mm ± 0.09, two albendazole = 1.9mm ± 0.11, Tukey HSD < 0.0001 for zero albendazole group). Taylor-Robinson conducted a meta-analysis of MUAC in four trials of children that were either all infected with STH or had high prevalence and received a single dose of deworming medication (Freij 1979a, Freij 1979b, Stephenson 1989, Stephenson 1993). The meta-analysis showed that children who received deworming medication had a significant mean difference in MUAC from children that received placebos [difference (95% CI): 0.49 cm (0.39–0.58)]. Taylor-Robinson conducted a meta-analysis of triceps skinfold thickness in three trials of children that were either all infected with STH or had high prevalence and received a single dose of deworming medication (Freij 1979a, Stephenson 1989, and Stephenson 1993). Children that received deworming medication had significantly greater mean triceps skinfold thickness than children in control groups [difference (95% CI): 1.34 mm (0.72–1.97)]. Taylor-Robinson conducted a meta-analysis of subscapular skinfold thickness in two trials of children that were either all infected with STH or had high prevalence and received a single dose of deworming medication (Stephenson 1989 and Stephenson 1993). Children that received deworming medication (174) had significantly greater mean subscapular skinfold thickness than children in control groups (172165 [difference (95% CI): 1.29 mm (1.13–1.44)].

One MDA trial [34] assessed the impact of deworming on change in knee-to-heel length over four months; no significant differences were found between treatment groups.

*Other measures* One treatment trial [51] and one MDA trial [34] investigated the impact of deworming on serum retinol concentration; neither found a significant difference between groups.

Two MDA trials [52] assessed the impact of deworming on infectious disease. Brutus 2007 reported that children aged 5-14 years, but not other age groups, treated with 3mg/kg of levimisole every two months for 18 months had significantly greater increase in *Plasmodium falciparum* parasite density than children given multivitamin tablets (one-way nested repeated-measures ANOVA = 0.58; 95% CI: 0.20 – 0.95, *P =* 0.003).

One MDA trial [53] investigated the impact of deworming on exercise induced bronchospasm; no significant differences were found between treatment groups.

Two MDA trials [31, 37] and one cross-sectional treatment study [44] assessed the impact of deworming on C-reactive protein; none found significant differences between groups.

One MDA trial [37] assessed the effect of deworming on soluble transferrin receptor (TfR). Rohner 2010 found that log TfR decreased significantly after anthelminthic treatment (log(TfR) = -0.14 mg/L, *P =* 0.03).

One cross-sectional treatment study [44] evaluated the effect of deworming on erythrocyte sedimentation rate. Karyadi 1996 found that among infected children, both those who received albendazole (*n* = 15) and those who received placebo (*n* = 19) had a significant increase in erythrocyte sedimentation rate (*P <* 0.05), however a MANOVA showed that there was a significant difference of the group and timing factors (pre-treatment treatment group mean 11.4 ± 9.0 mm/h, pre-treatment placebo group mean 10.8 ± 7.0 mm/h, post-treatment treatment group mean 17.3 ± 7.9 mm/h, post-treatment placebo group mean 17.8 ± 9.5 mm/h, *P <* 0.05).

One cross-sectional treatment study [44] assessed the effect of deworming on cytokine responses and tumor necrosis factor; no significant differences were found between treatment groups.

Two MDA trials [53, 54] investigated the impact of deworming on skin sensitivity to allergens. Flohr 2010 found that after 12 months children treated with albendazole had significantly increased allergenic skin sensitization to the three allergens tested (OR = 1.31, *P =* 0.03; 95% CI: 1.02 – 1.67). van den Biggelaar 2004 reported that a significantly greater cumulative proportion of children in the treatment group converted to a positive skin test (Hazard Ratio = 1.77; 95% CI: 0.35 – 0.90).

Two MDA trials [54, 55] assessed the effect of deworming on immunoglobulin levels (IgG). Cooper 2008 showed that albendazole receipt was associated with a significant reduction immunoglobulin-E (IgE) [OR (95% CI): 0.77 (0.7 – 0.86)].

***Women of reproductive age***

The following are additional outcomes that we investigated in women of reproductive age. See Supplementary Figure 3.

**Supplementary Figure 3. Evidence for an effect of deworming in women of reproductive age**

**

**

*Anemia-related measures* One treatment trial [56] assessed the effect of deworming on infant anemia; no significant differences were found.

One MDA trial [6] assessed the impact of maternal deworming on child hemoglobin during the first five years; no treatment effect of deworming was found overall or in a subgroup analysis of children whose mother was infected with hookworm.

One observational study [57] assessed the effect of deworming on maternal iron deficiency. Abel 2000 found that the prevalence of iron deficiency was significantly less in the study than the control area at a serum ferritin cutoff of <12 *µ*g/l (*P <*0.05) and <12 *µ*g/l (*P <*0.01), but the effect was not significant at higher cutoffs (serum ferritin <20 *µ*g/l and <24 *µ*g/l).

One MDA trial [58] and one observational study [59] assessed the impact of deworming on serum ferritin. Atukorala 1994 found that women who recalled taking an anthelminthic in their first trimester (*n* = 51) had significant increase in serum ferritin compared to women that did not recall treatment (*n* = 51)[Kruskal-Wallace test for two groups: *H* = 8.56, *P* < 0.005]. Torlesse 2001 found no significant differences in serum ferritin between groups.

*Anthropometric measures* One MDA trial [6] assessed child growth in children whose mothers were treated during pregnancy; no significant differences were found.

*Other Measures* Two observational studies [60, 61], two treatment trials [56, 62, 63], and one meta-analysis [63] assessed the effect of deworming on adverse birth outcomes (e.g., abortions, preterm deliveries, malformations). None of the studies found a significant treatment effect.

One MDA trial [64] and one observational MDA study [65] assessed the effect of deworming on congenital abnormalities; no significant differences were found.

One MDA trial [66] assessed the effect of deworming on infectious disease incidence and HIV indicators. Webb 2012 conducted a subgroup analysis of HIV-infected women from a previously reported study. HIV positive women who had been treated with deworming showed no significant difference in mean viral load in unadjusted analyses, but after adjustment for baseline viral load, asymptomatic malaria, and viral load at six weeks a marginal difference was found [adj. mean difference (95% CI): 0.24 log_10_ (0.01–0.47), *P =* 0.03].

One MDA trial [6] assessed cognitive outcomes, eczema, and allergic responses in children of women who were treated with deworming medications. Ndibazza 2012 showed that maternal albendazole receipt was associated with marginally significantly lower executive function scores as measured with the Wisconsin Card-Sorting Test [*ß* (95% CI): -0.54 (-1.07– -0.01), *P =* 0.05] and higher rates of eczema [HR (95% CI): 1.58 (1.15–2.17), *P =* 0.005]. No significant differences were found in measures of allergic responses.

***Meta-analysis***

*Mortality* We used estimates for effect of deworming children younger than five years on mortality from six studies (Awasthi 2000, Awasthi 2001, Awasthi 2008, Awasthi 2013, Donnen 1998, and Ndibazza 2012) [1-6]. The log-RR of death between treatment and control groups varied from -0.26 to 0.26 (Supplementary Fig. 4). In a random-effect model the pooled log RR (95% CI) for treatment versus control was -0.02 (-0.14–0.10, *P* = 0.713). There was little evidence of heterogeneity (*I^2^* = 29.9%, Cochrane’s *Q* = 6.28, *P* = 0.280). These results were robust to leaving any study out; however, leaving Awasthi 2001 out lead to a reduction in heterogeneity (*I^2^* = 0.0%) and to a marginally significant result favoring control groups [log RR (95% CI): -0.06 (0.13–0.01), *P* = 0.095].

**Supplementary Figure 4**

**
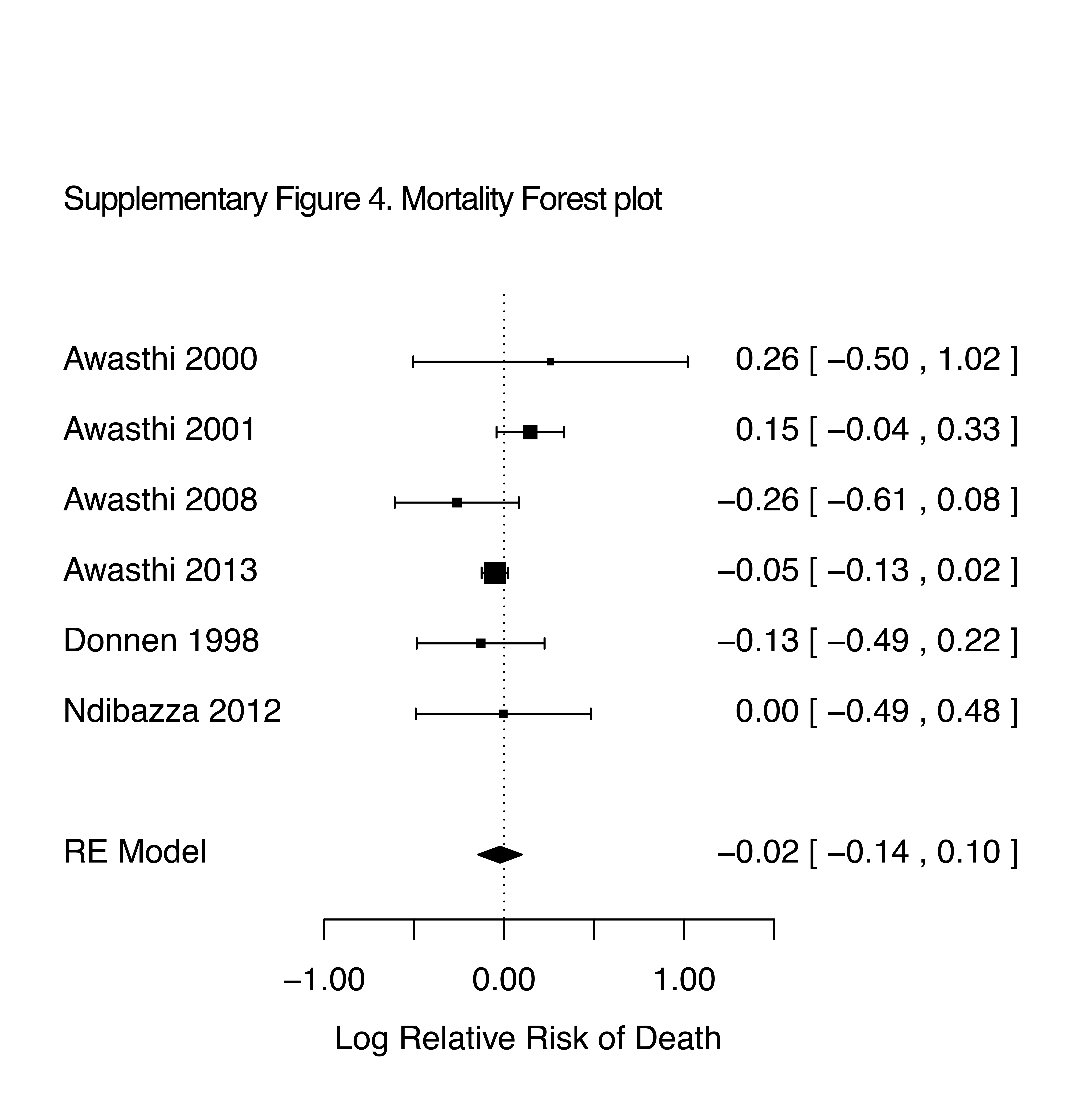
**

*Hemoglobin* We used estimates for the effect of deworming children younger than five years on hemoglobin from nine studies (Awasthi 2000, Dossa 2001, Garg 2002, Goto 2009, Kirwan 2010, Ndibazza 2012, Palupi 1997, Stoltzfus 2001, Tanumihardjo 2004) [2, 6-8, 10, 12, 15, 26, 67]. The estimates of mean difference ranged from -0.30 to 0.30 (Supplementary Fig. 5). In a random-effect model the pooled mean difference (95% CI) for treatment versus control was -0.01 (-0.07–0.05, *P* = 0.766). There was little evidence of heterogeneity (*I^2^* = 0.0%, Cochrane’s *Q* = 4.13, *P* = 0.903). These results were robust to leaving any study out.

**Supplementary Figure 5**

**
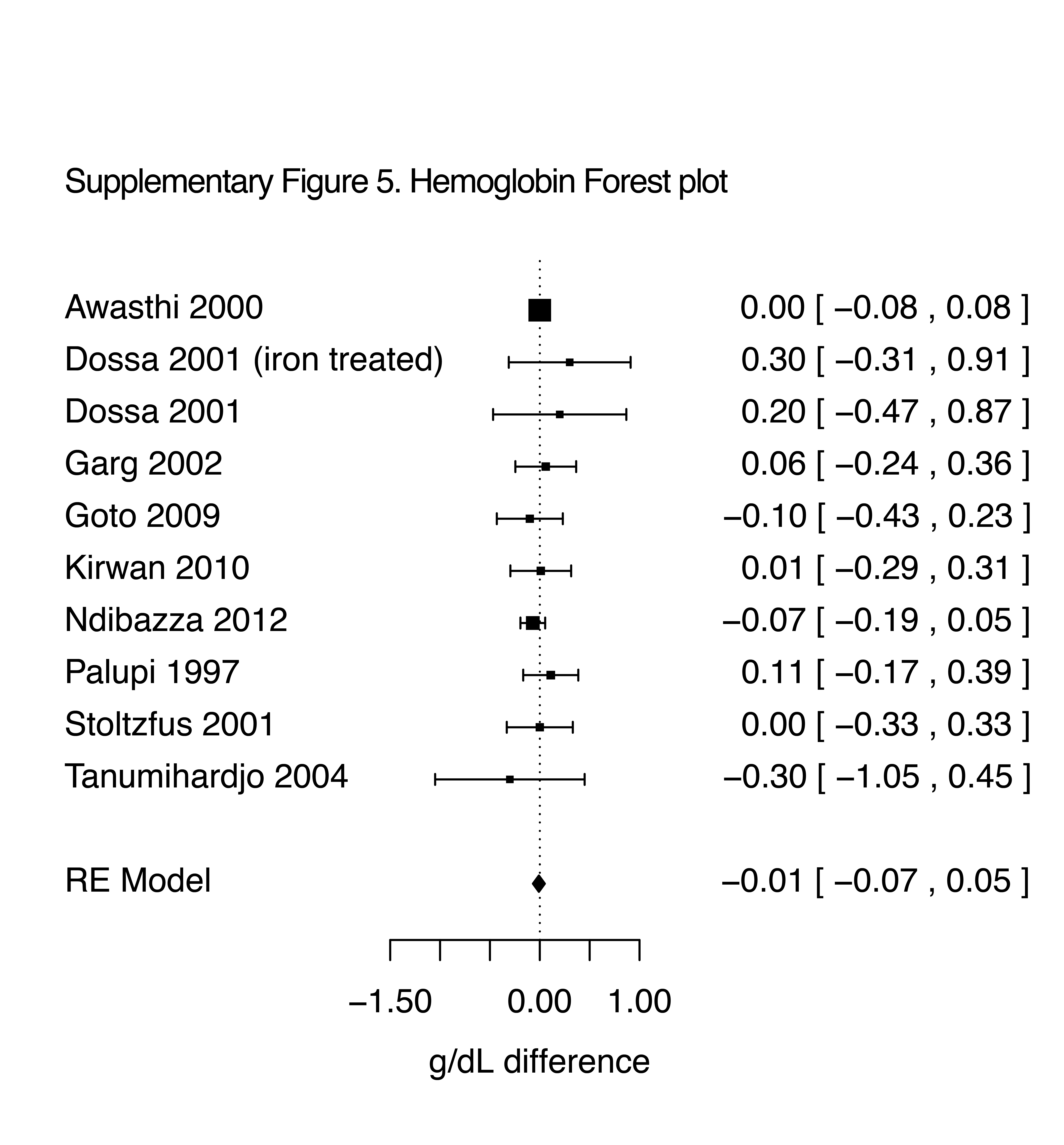
**

*Height* We used estimates for the effect of deworming children younger than five years on hemoglobin from nine studies (Awasthi 2000, Awasthi 2001, Awasthi 2008, Donnen 1998, Dossa 2001, Garg 2002, Gupta 1982, Joseph 2015, and Palupi 1997) [1-3, 5, 7, 8, 15, 16, 18]. The estimates of mean difference ranged from -1.19 to 0.50 (Supplementary Fig. 6). In a random-effect model the pooled mean difference (95% CI) for treatment versus control was -0.03 (-0.19–0.12). There was little evidence heterogeneity (*I^2^* = 7.38%, Cochrane’s *Q* = 12.60, *p* = 0.25). These results were robust to leaving any single study out.

**Supplementary Figure 6**

**
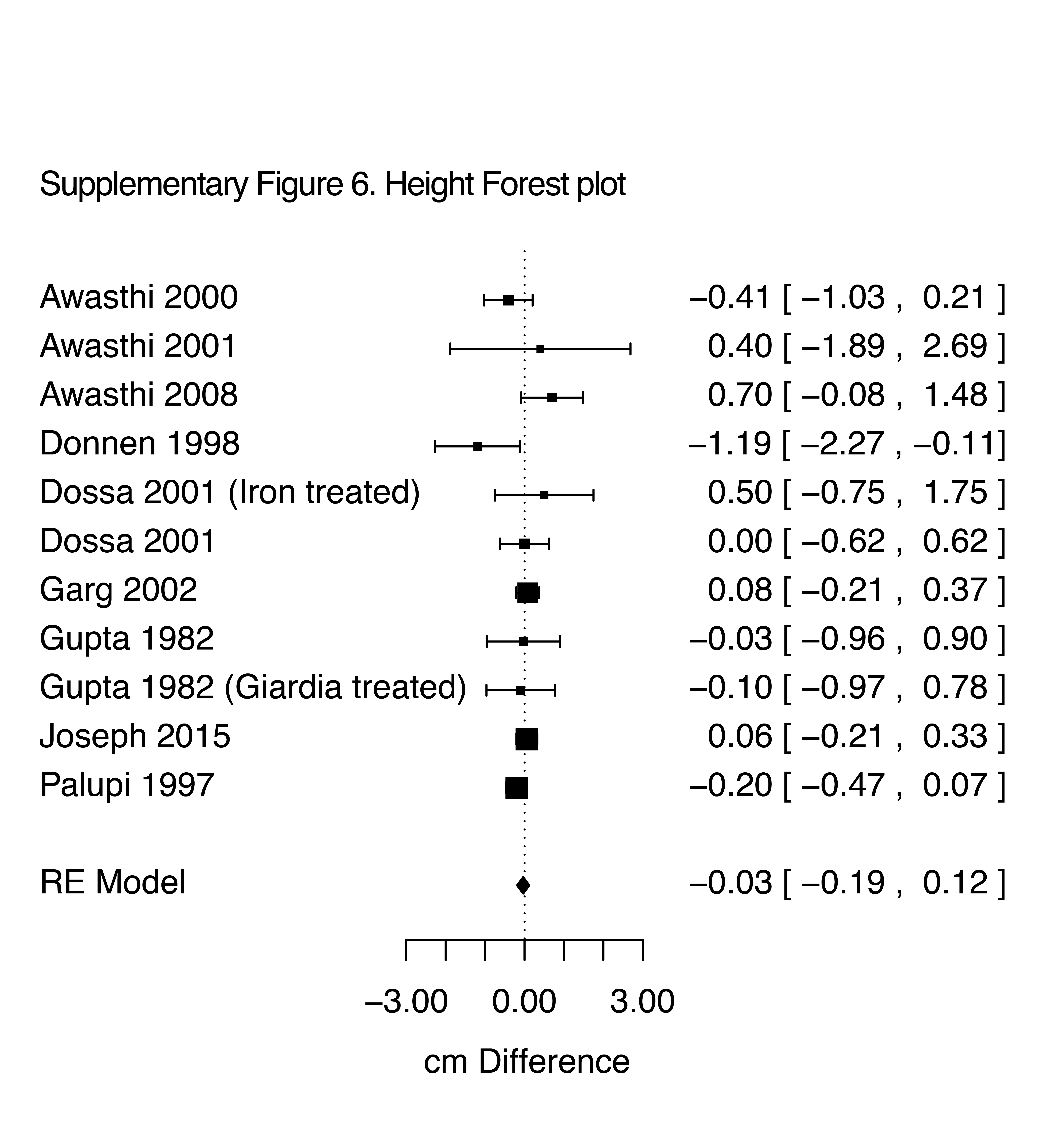
**

*HAZ* We used estimates for the effect of deworming children younger than five years on HAZ from seven studies (Awasthi 2001, Dossa 2001, Garg 2002, Joseph 2015, Ndibazza 2012, Northrop-Clewes 2001, and Palupi 1997) [1, 6-8, 11, 15, 18]. The estimates of mean difference ranged from -0.01 to 0.12 (Supplementary Fig. 7). In a random-effect model the pooled mean difference (95% CI) for treatment versus control was -0.01 (-0.06–0.03). There was little evidence for heterogeneity (*I^2^* = 0.01%, Cochrane’s *Q* = 8.64, *P* = 0.279). These results were robust to leaving any single study out.

**Supplementary Figure 7**

**
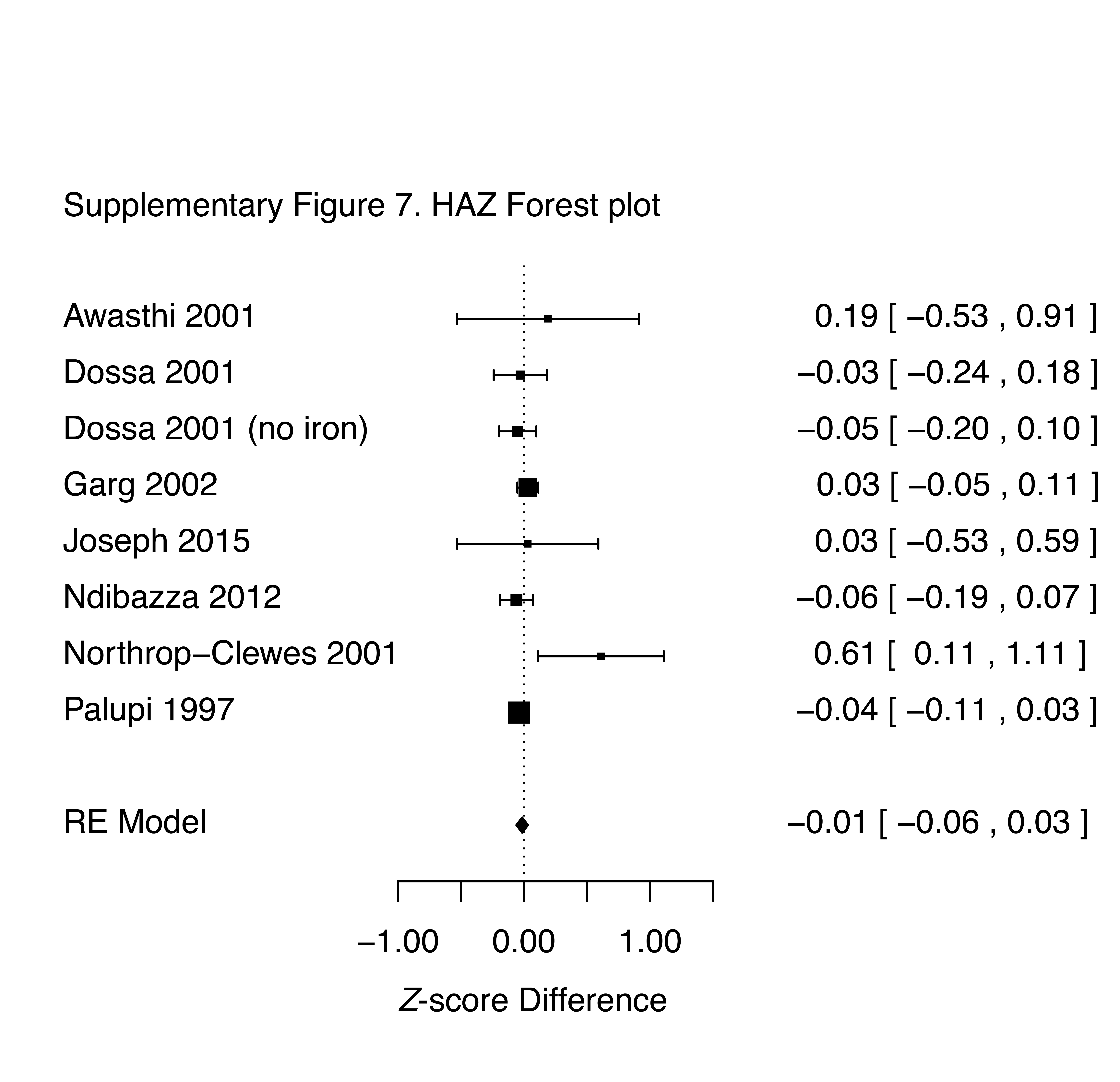
**

*WAZ* We used estimates for the effect of deworming children younger than five years on WAZ from six studies (Awasthi 2001, Garg 2002, Joseph 2015, Ndibazza 2012, Northrop-Clewes 2001, and Palupi 1997) [1, 6, 8, 11, 15, 18]. The estimates of mean difference ranged from -0.05 to 0.61 (Supplementary Fig. 9). In a random-effect model the pooled mean difference (95% CI) for treatment versus control was -0.00 (-0.03–0.02). There was little evidence for heterogeneity (*I^2^* = 5.58%, Cochrane’s *Q* = 2.38, *P* = 0.795). In our leave-one-out analysis, the results became significantly negative [pooled mean difference (95% CI): -0.01 (-0.02– -0.00)] when Joseph 2015 was removed.

**Supplementary Figure 8**

**
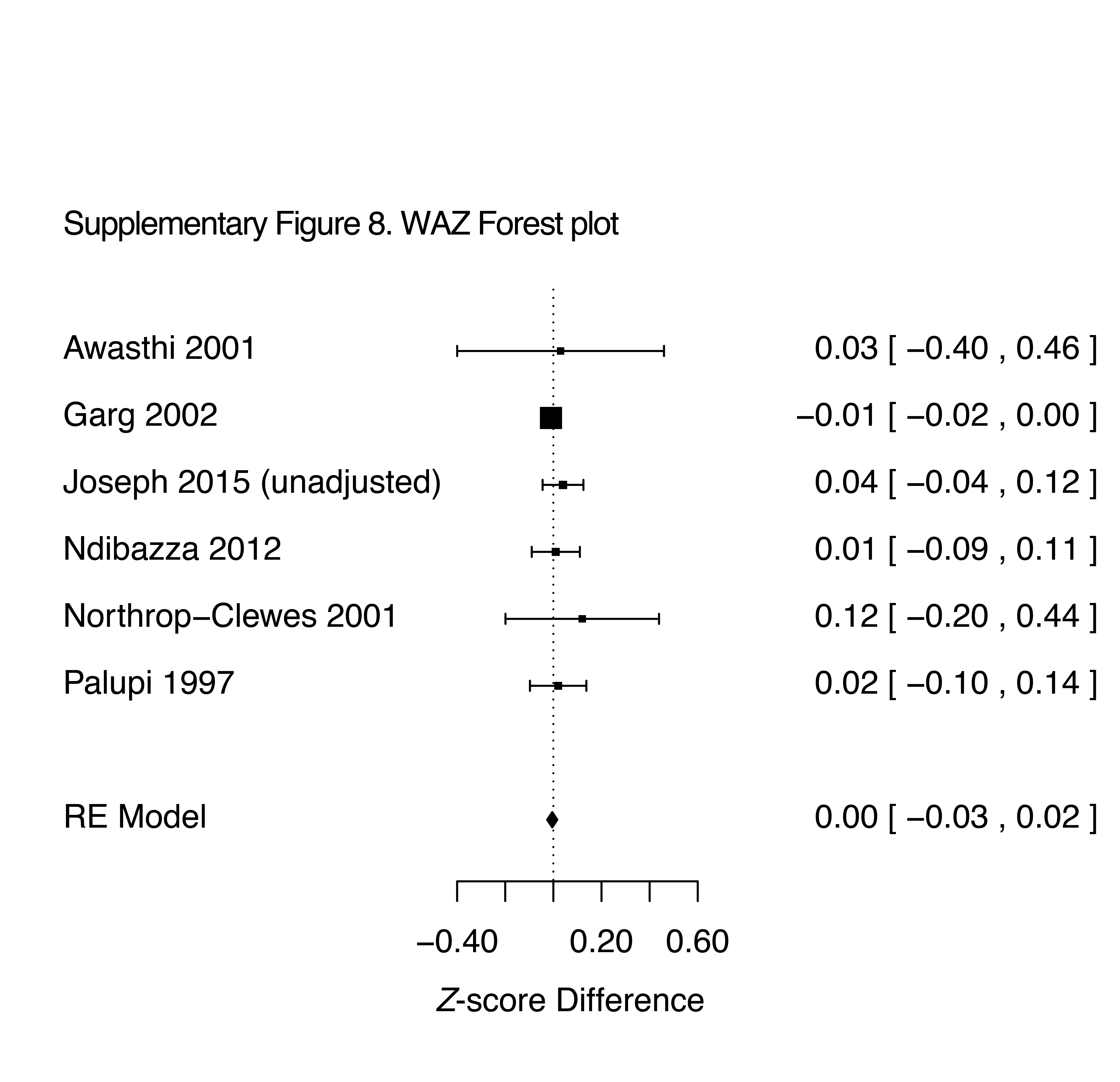
**

*WHZ* We used estimates for the effect of deworming children younger than five years on WHZ from four studies (Awasthi 2001, Dossa 2001, Garg 2002, and Ndibazza 2012) [1, 6, 7, 15]. The estimates of mean difference ranged from -0.15 to 0.04 (Supplementary Fig. 9). In a random-effect model the pooled mean difference (95% CI) for treatment versus control was -0.01 (-0.08–0.07). There was little evidence for heterogeneity (*I^2^* = 0.00%, Cochrane’s *Q* = 1.72, *P* = 0.787). These results were robust to leaving any single study out.

**Supplementary Figure 9**

**
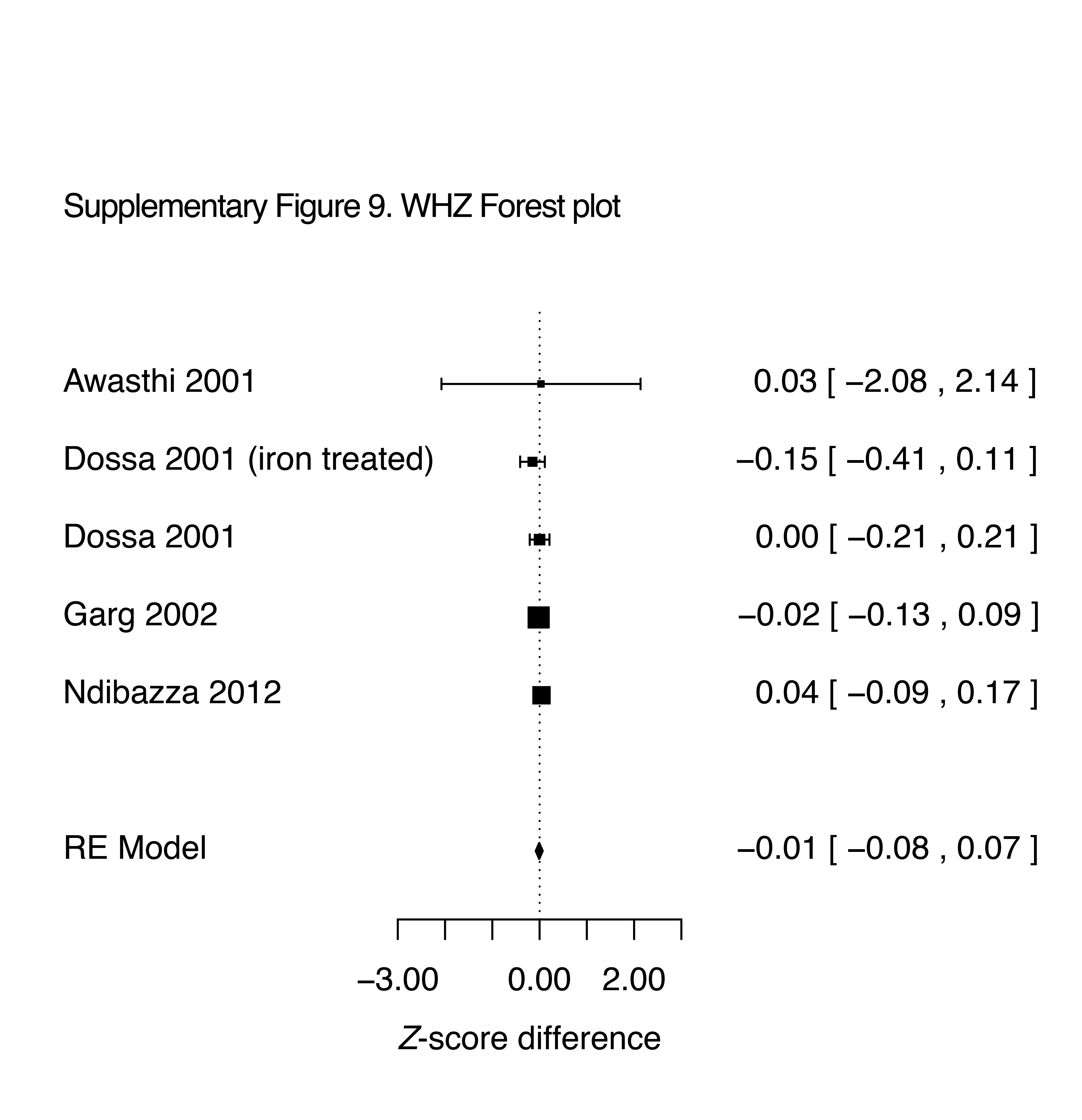
**

*MUAC* We used estimates for the effect of deworming children younger than five years on WHZ from four studies (Donnen 1998, Dossa 2001, Freij 1979a, Freij 1979b, and Northrop-Clewes 2001) [5, 7, 11, 21]. The estimates of mean difference ranged from -0.35 to 0.40 (Supplementary Fig. 10). In a random-effect model the pooled mean difference (95% CI) for treatment versus control was 0.02 (-0.25–0.29). There was some evidence of heterogeneity (*I^2^* = 51.61%, Cochrane’s *Q* = 10.09, *P* = 0.073). These results were robust to leaving any single study out; when Donnen 1998 was removed the heterogeneity was substantially reduced (*I^2^* = 0.00%).

**Supplementary Figure 10**

**
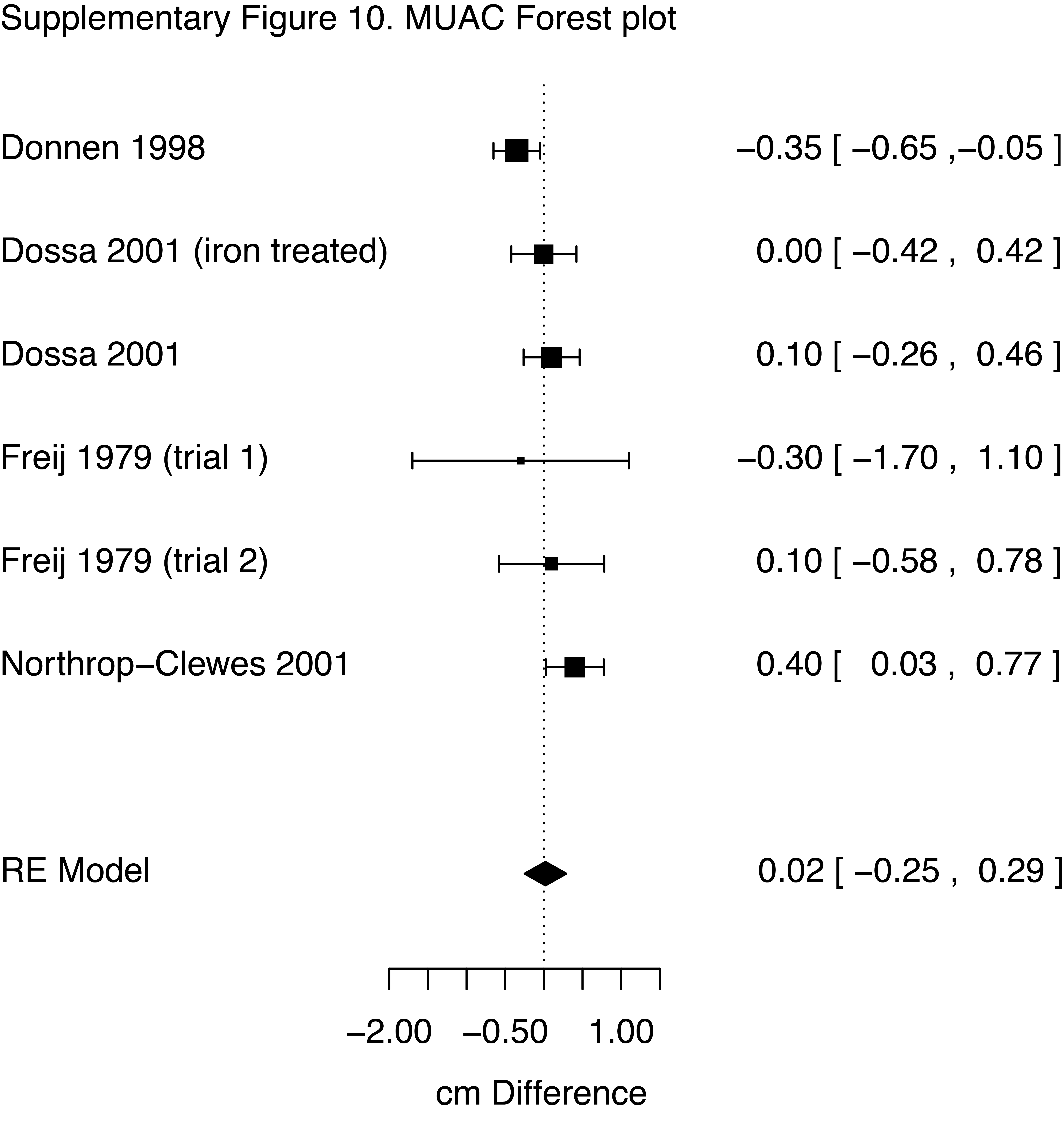
**

1. Awasthi S, Pande VK: **Six-monthly de-worming in infants to study effects on growth**. *The Indian Journal of Pediatrics* 2001, **68**(9):823-827.

2. Awasthi S, Pande VK, Fletcher RH: **Effectiveness and cost-effectivenss of albendazole in improving nutritional status of pre-school children in urban slums**. *Indian pediatrics* 2000, **37**(1):19-30.

3. Awasthi S, Peto R, Pande VK, Fletcher RH, Read S, Bundy DA: **Effects of deworming on malnourished preschool children in India: an open-labelled, cluster-randomized trial**. *PLoS Negl Trop Dis* 2008, **2**(4):e223.

4. Awasthi S, Peto R, Read S, Richards SM, Pande V, Bundy D: **Population deworming every 6 months with albendazole in 1 million pre-school children in north India: DEVTA, a cluster-randomised trial**. *The Lancet* 2013, **381**(9876):1478-1486.

5. Donnen P, Brasseur D, Dramaix M, Vertongen F, Zihindula M, Muhamiriza M, Hennart P: **Vitamin A supplementation but not deworming improves growth of malnourished preschool children in eastern Zaire**. *The Journal of nutrition* 1998, **128**(8):1320-1327.

6. Ndibazza J, Mpairwe H, Webb EL, Mawa PA, Nampijja M, Muhangi L, Kihembo M, Lule SA, Rutebarika D, Apule B: **Impact of anthelminthic treatment in pregnancy and childhood on immunisations, infections and eczema in childhood: a randomised controlled trial**. *PloS one* 2012, **7**(12):e50325.

7. Dossa R, Ategbo E, De Koning F, Van Raaij J, Hautvast J: **Impact of iron supplementation and deworming on growth performance in preschool Beninese children**. *European Journal of Clinical Nutrition* 2001, **55**(4):223-228.

8. Palupi L, Schultink W, Achadi E, Gross R: **Effective community intervention to improve hemoglobin status in preschoolers receiving once-weekly iron supplementation**. *The American journal of clinical nutrition* 1997, **65**(4):1057-1061.

9. Stoltzfus RJ, Chway HM, Montresor A, Tielsch JM, Jape JK, Albonico M, Savioli L: **Low dose daily iron supplementation improves iron status and appetite but not anemia, whereas quarterly anthelminthic treatment improves growth, appetite and anemia in Zanzibari preschool children**. *The Journal of nutrition* 2004, **134**(2):348-356.

10. Stoltzfus RJ, Kvalsvig JD, Chwaya HM, Montresor A, Albonico M, Tielsch JM, Savioli L, Pollitt E: **Effects of iron supplementation and anthelmintic treatment on motor and language development of preschool children in Zanzibar: double blind, placebo controlled study**. *Bmj* 2001, **323**(7326):1389.

11. Northrop-Clewes CA, Rousham EK, Mascie-Taylor CN, Lunn PG: **Anthelmintic treatment of rural Bangladeshi children: effect on host physiology, growth, and biochemical status**. *The American journal of clinical nutrition* 2001, **73**(1):53-60.

12. Goto R, Mascie-Taylor CN, Lunn PG: **Impact of anti-Giardia and anthelminthic treatment on infant growth and intestinal permeability in rural Bangladesh: a randomised double-blind controlled study**. *Transactions of the Royal Society of Tropical Medicine and Hygiene* 2009, **103**(5):520-529.

13. Rousham E, Mascie-Taylor C: **An 18-month study of the effect of periodic anthelminthic treatment on the growth and nutritional status of pre-school children in Bangladesh**. *Annals of human biology* 1994, **21**(4):315-324.

14. Northrop C, Lunn P, Wainwright M, Evans J: **Plasma albumin concentrations and intestinal permeability in Bangladeshi children infected with Ascaris lumbricoides**. *Transactions of the Royal Society of Tropical Medicine and Hygiene* 1987, **81**(5):811-815.

15. Garg R, Lee LA, Beach MJ, Wamae C, Ramakrishnan U, Deming MS: **Evaluation of the integrated management of childhood illness guidelines for treatment of intestinal helminth infections among sick children aged 2–4 years in western Kenya**. *Transactions of the Royal Society of Tropical Medicine and Hygiene* 2002, **96**(5):543-548.

16. Gupta MC, Urrutia JJ: **Effect of periodic antiascaris and antigiardia treatment on nutritional status of preschool children**. *The American Journal of Clinical Nutrition* 1982, **36**(1):79-86.

17. Willett WC, Kilama W, Kihamia C: **Ascaris and growth rates: a randomized trial of treatment**. *American Journal of Public Health* 1979, **69**(10):987-991.

18. Joseph SA, Casapía M, Montresor A, Rahme E, Ward BJ, Marquis GS, Pezo L, Blouin B, Maheu-Giroux M, Gyorkos TW: **The effect of deworming on growth in one-year-old children living in a soil-transmitted helminth-endemic area of Peru: a randomized controlled trial**. *PLoS Negl Trop Dis* 2015, **9**(10):e0004020.

19. Greenberg B, Gilman RH, Shapiro H, Gilman J, Mondal G, Maksud M, Khatoon H, Chowdhury J: **Single dose piperazine therapy for Ascaris lumbricoides: an unsuccessful method of promoting growth**. *The American journal of clinical nutrition* 1981, **34**(11):2508-2516.

20. Kloetzel K, MERLUZZI FILHO TJ, KLOETZEL D: **Ascaris and malnutrition in a group of Brazilian children—a follow-up study**. *Journal of tropical pediatrics* 1982, **28**(1):41-43.

21. Freij L, Meeuwisse GW, Berg NO, Wall S, Gebre-Medhin M: **Ascariasis and malnutrition. A study in urban Ethiopian children**. *The American journal of clinical nutrition* 1979, **32**(7):1545-1553.

22. Stephenson L, Crompton D, Latham M, Schulpen T, Nesheim M, Jansen A: **Relationships between Ascaris infection and growth of malnourished preschool children in Kenya**. *The American journal of clinical nutrition* 1980, **33**(5):1165-1172.

23. Jalal F, Nesheim M, Agus Z, Sanjur D, Habicht J: **Serum retinol concentrations in children are affected by food sources of beta-carotene, fat intake, and anthelmintic drug treatment**. *The American journal of clinical nutrition* 1998, **68**(3):623-629.

24. Tanumihardjo SA, Permaesih D, Rustan E, Rusmil K: **Vitamin A status of Indonesian children infected with Ascaris lumbricoides after dosing with vitamin A supplements and albendazole**. *The Journal of nutrition* 1996, **126**(2):451.

25. Reddy V, Vijayaraghavan K, Mathur KK: **Effect of deworming and vitamin A administration on serum vitamin A levels in preschool children**. *Journal of tropical pediatrics* 1986, **32**(4):196-199.

26. Kirwan P, Jackson AL, Asaolu SO, Molloy SF, Abiona TC, Bruce MC, Ranford-Cartwright L, MO'Neill S, Holland CV: **Impact of repeated four-monthly anthelmintic treatment on Plasmodium infection in preschool children: a double-blind placebo-controlled randomized trial**. *BMC infectious diseases* 2010, **10**(1):1.

27. Croke K, Hicks JH, Hsu E, Kremer M, Miguel E: **Does Mass Deworming Affect Child Nutrition? Meta-analysis, Cost-Effectiveness, and Statistical Power**. In*.*: National Bureau of Economic Research; 2016.

28. Taylor‐Robinson DC, Maayan N, Soares‐Weiser K, Donegan S, Garner P: **Deworming drugs for soil‐transmitted intestinal worms in children: effects on nutritional indicators, haemoglobin, and school performance**. *The Cochrane Library* 2015.

29. Aiken AM, Davey C, Hargreaves JR, Hayes RJ: **Re-analysis of health and educational impacts of a school-based deworming programme in western Kenya: a pure replication**. *International journal of epidemiology* 2015, **44**(5):1572-1580.

30. Davey C, Aiken AM, Hayes RJ, Hargreaves JR: **Re-analysis of health and educational impacts of a school-based deworming programme in western Kenya: a statistical replication of a cluster quasi-randomized stepped-wedge trial**. *International journal of epidemiology* 2015:dyv128.

31. Bhargava A, Jukes M, Lambo J, Kihamia C, Lorri W, Nokes C, Drake L, Bundy D: **Anthelmintic treatment improves the hemoglobin and serum ferritin concentrations of Tanzanian schoolchildren**. *Food and nutrition bulletin* 2003, **24**(4):332-342.

32. Bhoite RM, Iyer UM: **Effect of deworming vs iron-folic acid supplementation plus deworming on growth, hemoglobin level, and physical work capacity of schoolchildren**. *Indian pediatrics* 2012, **49**(8):659-661.

33. Ebenezer R, Gunawardena K, Kumarendran B, Pathmeswaran A, Jukes MC, Drake LJ, Silva N: **Cluster‐randomised trial of the impact of school‐based deworming and iron supplementation on the cognitive abilities of schoolchildren in Sri Lanka's plantation sector**. *Tropical Medicine & International Health* 2013, **18**(8):942-951.

34. Jinabhai CC, Taylor M, Coutsoudis A, Coovadia HM, Tomkins AM, Sullivan KR: **A randomized controlled trial of the effect of antihelminthic treatment and micronutrient fortification on health status and school performance of rural primary school children**. *Annals of Tropical Paediatrics: International Child Health* 2001, **21**(4):319-333.

35. Le HT, Brouwer ID, Nguyen KC, Burema J, Kok FJ: **The effect of iron fortification and de-worming on anaemia and iron status of Vietnamese schoolchildren**. *British Journal of Nutrition* 2007, **97**(05):955-962.

36. Nga TT, Winichagoon P, Dijkhuizen MA, Khan NC, Wasantwisut E, Furr H, Wieringa FT: **Multi-micronutrient–fortified biscuits decreased prevalence of anemia and improved micronutrient status and effectiveness of deworming in rural Vietnamese school children**. *The Journal of nutrition* 2009, **139**(5):1013-1021.

37. Rohner F, Zimmermann MB, Amon RJ, Vounatsou P, Tschannen AB, N'Goran EK, Nindjin C, Cacou M-C, Té-Bonlé MD, Aka H: **In a randomized controlled trial of iron fortification, anthelmintic treatment, and intermittent preventive treatment of malaria for anemia control in Ivorian children, only anthelmintic treatment shows modest benefit**. *The Journal of nutrition* 2010, **140**(3):635-641.

38. Taylor M, Jinabhai C, Couper I, Kleinschmidt I, Jogessar V: **The effect of different anthelmintic treatment regimens combined with iron supplementation on the nutritional status of schoolchildren in KwaZulu-Natal, South Africa: a randomized controlled trial**. *Transactions of the Royal Society of Tropical Medicine and Hygiene* 2001, **95**(2):211-216.

39. Miguel E, Kremer M: **Worms: identifying impacts on education and health in the presence of treatment externalities**. *Econometrica* 2004, **72**(1):159-217.

40. Sufiyan M, Sabitu K, Mande A: **Evaluation of the effectiveness of deworming and participatory hygiene education strategy in controlling anemia among children aged 6-15 years in Gadagau community, Giwa LGA, Kaduna, Nigeria**. *Annals of African medicine* 2011, **10**(1).

41. Adams EJ, Stephenson LS, Latham MC, Kinoti SN: **Physical activity and growth of Kenyan school children with hookworm, Trichuris trichiura and Ascaris lumbricoides infections are improved after treatment with albendazole**. *The Journal of nutrition* 1994, **124**(8):1199-1206.

42. Friis H, Mwaniki D, Omondi B, Muniu E, Thiong'o F, Ouma J, Magnussen P, Geissler P, Michaelsen KF: **Effects on haemoglobin of multi-micronutrient supplementation and multi-helminth chemotherapy: a randomized, controlled trial in Kenyan school children**. *European journal of clinical nutrition* 2003, **57**(4):573-579.

43. Kruger M, Badenhorst C, Mansvelt E, Laubscher J, Benadé AS: **Effects of iron fortification in a school feeding scheme and anthelmintic therapy on the iron status and growth of six-to eight-year-old schoolchildren**. 1996.

44. Karyadi E, Gross R, Sastroamidjojo S, Dillon D, Richards A, Sutanto I: **Anthelminthic treatment raises plasma iron levels but does not decrease the acute-phase response in Jakarta school children**. *Southeast Asian journal of tropical medicine and public health* 1996, **27**:742-753.

45. Tee MH, Lee YY, Majid NA, Noori NM, Raj SM: **Growth reduction among primary school-children with light trichuriasis in Malaysia treated with albendazole**. *Southeast Asian J Trop Med Public Health* 2013, **44**(1):19-24.

46. Yap P, Wu F-W, Du Z-W, Hattendorf J, Chen R, Jiang J-Y, Kriemler S, Krauth SJ, Zhou X-N, Utzinger J: **Effect of deworming on physical fitness of school-aged children in Yunnan, China: a double-blind, randomized, placebo-controlled trial**. *PLoS Negl Trop Dis* 2014, **8**(7):e2983.

47. Beach MJ, Streit TG, Addiss DG, Prospere R, Roberts JM, Lammie PJ: **Assessment of combined ivermectin and albendazole for treatment of intestinal helminth and Wuchereria bancrofti infections in Haitian schoolchildren**. *The American journal of tropical medicine and hygiene* 1999, **60**(3):479-486.

48. Fox LM, Furness BW, Haser JK, Desire D, Brissau J-m, Milord M-d, Lafontant J, Lammie PJ, Beach MJ: **Tolerance and efficacy of combined diethylcarbamazine and albendazole for treatment of Wuchereria bancrofti and intestinal helminth infections in Haitian children**. *The American journal of tropical medicine and hygiene* 2005, **73**(1):115-121.

49. Stephenson LS, Latham MC, Adams EJ, Kinoti SN, Pertet A: **Weight gain of Kenyan school children infected with hookworm, Trichuris trichiura and Ascaris lumbricoides is improved following once-or twice-yearly treatment with albendazole**. *The Journal of nutrition* 1993, **123**(4):656-665.

50. Watkins WE, Cruz JR, Pollitt E: **The effects of deworming on indicators of school performance in Guatemala**. *Transactions of the Royal Society of Tropical Medicine and Hygiene* 1996, **90**(2):156-161.

51. Mwaniki D, Omondi B, Muniu E, Thiong'o F, Ouma J, Magnussen P, Geissler P, Michaelsen K, Friis H: **Effects on serum retinol of multi-micronutrient supplementation and multi-helminth chemotherapy: a randomised, controlled trial in Kenyan school children**. *European journal of clinical nutrition* 2002, **56**(7):666-673.

52. Brutus L, Watier L, Hanitrasoamampionona V, Razanatsoarilala H, Cot M: **Confirmation of the protective effect of Ascaris lumbricoides on Plasmodium falciparum infection: results of a randomized trial in Madagascar**. *The American journal of tropical medicine and hygiene* 2007, **77**(6):1091-1095.

53. Flohr C, Tuyen L, Quinnell R, Lewis S, Minh T, Campbell J, Simmons C, Telford G, Brown A, Hien T: **Reduced helminth burden increases allergen skin sensitization but not clinical allergy: a randomized, double‐blind, placebo‐controlled trial in Vietnam**. *Clinical & Experimental Allergy* 2010, **40**(1):131-142.

54. van den Biggelaar AH, Rodrigues LC, van Ree R, van der Zee JS, Hoeksma-Kruize YC, Souverijn JH, Missinou MA, Borrmann S, Kremsner PG, Yazdanbakhsh M: **Long-term treatment of intestinal helminths increases mite skin-test reactivity in Gabonese schoolchildren**. *Journal of Infectious Diseases* 2004, **189**(5):892-900.

55. Cooper PJ, Alexander N, Moncayo A-L, Benitez SM, Chico ME, Vaca MG, Griffin GE: **Environmental determinants of total IgE among school children living in the rural Tropics: importance of geohelminth infections and effect of anthelmintic treatment**. *BMC immunology* 2008, **9**(1):1.

56. Ndyomugyenyi R, Kabatereine N, Olsen A, Magnussen P: **Efficacy of ivermectin and albendazole alone and in combination for treatment of soil-transmitted helminths in pregnancy and adverse events: a randomized open label controlled intervention trial in Masindi district, western Uganda**. *The American journal of tropical medicine and hygiene* 2008, **79**(6):856-863.

57. Abel R, Rajaratnam J, Kalaimani A, Kirubakaran S: **Can iron status be improved in each of the three trimesters? A community-based study**. *European journal of clinical nutrition* 2000, **54**(6):490-493.

58. Torlesse H, Hodges M: **Albendazole therapy and reduced decline in haemoglobin concentration during pregnancy (Sierra Leone)**. *Transactions of the Royal Society of Tropical Medicine and Hygiene* 2001, **95**(2):195-201.

59. Atukorala T, De Silva L, Dechering W, Dassenaeike T, Perera RS: **Evaluation of effectiveness of iron-folate supplementation and anthelminthic therapy against anemia in pregnancy--a study in the plantation sector of Sri Lanka**. *The American journal of clinical nutrition* 1994, **60**(2):286-292.

60. Adam I, Homeida M: **Is praziquantel therapy safe during pregnancy?** *Transactions of the Royal Society of Tropical Medicine and Hygiene* 2004, **98**(9):540-543.

61. Passerini L, Casey GJ, Biggs BA, Cong DT, Phu LB, Phuc TQ, Carone M, Montresor A: **Increased birth weight associated with regular pre-pregnancy deworming and weekly iron-folic acid supplementation for Vietnamese women**. *PLoS Negl Trop Dis* 2012, **6**(4):e1608.

62. Gyorkos TW, Larocque R, Casapia M, Gotuzzo E: **Lack of risk of adverse birth outcomes after deworming in pregnant women**. *The Pediatric infectious disease journal* 2006, **25**(9):791-794.

63. Salam RA, Haider BA, Humayun Q, Bhutta ZA: **Effect of administration of antihelminthics for soil‐transmitted helminths during pregnancy**. *The Cochrane Library* 2015.

64. Elliott AM, Ndibazza J, Mpairwe H, Muhangi L, Webb EL, Kizito D, Mawa P, Tweyongyere R, Muwanga M: **Treatment with anthelminthics during pregnancy: what gains and what risks for the mother and child?** *Parasitology* 2011, **138**(12):1499-1507.

65. De Silva N, Sirisena J, Gunasekera D, Ismail M, De Silva H: **Effect of mebendazole therapy during pregnancy on birth outcome**. *The Lancet* 1999, **353**(9159):1145-1149.

66. Webb EL, Kyosiimire-Lugemwa J, Kizito D, Nkurunziza P, Lule S, Muhangi L, Muwanga M, Kaleebu P, Elliott AM: **The effect of anthelminthic treatment during pregnancy on HIV plasma viral load; results from a randomised, double blinded, placebo-controlled trial in Uganda**. *Journal of acquired immune deficiency syndromes (1999)* 2012, **60**(3):307.

67. Tanumihardjo S, Permaesih D: **Vitamin A status and hemoglobin concentrations are improved in Indonesian children with vitamin A and deworming interventions**. *European journal of clinical nutrition* 2004, **58**(9):1223-1230.
